# The Dual‐Function of CtrNAC019‐CtrNPF2.1 Module in Salt Tolerance and Nitrogen Use Efficiency Via Enhancing Vacuolar Chloride Sequestration and Nitrate Efflux in *Citrus trifoliata*

**DOI:** 10.1111/pbi.70686

**Published:** 2026-05-18

**Authors:** Zeqi Zhao, Chengwei Yang, Xiangming Shang, Mengdi Li, Xiaoyang Su, Ji‐Hong Liu, Chunlong Li

**Affiliations:** ^1^ National Key Laboratory for Germplasm Innovation & Utilization of Horticultural Crops, College of Horticulture and Forestry Sciences Huazhong Agricultural University Wuhan China; ^2^ Hubei Hongshan Laboratory Wuhan China

**Keywords:** chloride, *Citrus trifoliata*, CtrNPF2.1, nitrogen‐use efficiency, salt stress

## Abstract

Salt stress and nitrogen utilisation efficiency (NUE) represent critical constraints affecting worldwide crop productivity. While nitrate transporter proteins (NPFs) have been implicated in saline chloride (Cl^−^) ion transport, the mechanistic linkage between chloride stress and NUE remains poorly understood. Through comparative transcriptomic profiling of *Citrus trifoliata* , we identified *CtrNPF2.1* as a dual‐responsive gene significantly upregulated in root tissues under both chloride salt and high nitrate conditions. The CtrNPF2.1 protein is localized to the vacuole membrane and highly expressed in root cortical cells. Transport functional characterisation combined with transgenic phenotypic analyses established that CtrNPF2.1 mediates dual transport mechanisms: facilitating vacuolar Cl^−^ compartmentalisation while enabling nitrate efflux. This coordinated process enhances cellular ion detoxification and promotes nitrate redistribution for optimal root development under saline conditions. Molecular investigations further uncovered that CtrNAC019 transcriptionally regulates *CtrNPF2.1* expression through direct binding to its promoter, forming a chloride/nitrate‐responsive regulatory module. Notably, physiological validation reveals coordinated responses in which nitrate supplementation mitigates chloride toxicity while chloride availability enhances nitrogen utilisation in trifoliate orange, which may be explained by the CtrNAC019‐CtrNPF2.1 expression regulation module. These results provide new insights into vacuolar chloride sequestration and nitrate efflux by CtrNAC019‐CtrNPF2.1 under saline and high‐nitrate conditions, offering a promising strategy for breeding salt‐resilient and resource‐efficient crops.

## 1 Introduction

Saline stress is an unavoidable abiotic stress that adversely impedes plant growth and crop yield globally (Zelm et al. 2020; Zhang and Huang 2019). Unlike other abiotic stresses, plants inevitably accumulate substantial amounts of salt ions under salinity conditions, typically including sodium and chloride ions. Once the ion concentration exceeds the threshold within the plant body, metabolic blockage occurs, leading to disruption of normal physiological activities and accumulation of excessive reactive oxygen species (ROS), thus causing cellular damage (Yang and Guo 2018; Zhao et al. 2018). It has been reported that excessive Na^+^ disrupts the homeostasis of sodium and potassium ions, thereby affecting cellular metabolism and ultimately inhibiting plant growth and development (Hualpa‐Ramirez et al. 2024; Katiyar‐Agarwal et al. 2006). In addition, substantial chloride can lead to the structural disruption of chloroplasts, increased plasma membrane permeability, and nutrient depletion (Ren et al. 2021). However, the chloride ion is often neglected compared to the sodium ion in salt stress‐related biological studies.

Through evolution and natural selection, plants have developed a diverse array of sophisticated mechanisms to alleviate the detrimental effects of salt stress. These mechanisms include the scavenging of ROS, the accumulation of osmoregulatory compounds, and the regulation of salt ion transport. Among them, the modulation of salt ion transport can directly mitigate the toxicity resulting from ion accumulation, serving as an essential process for enhancing salt tolerance in plants (Zhu 2002; Zhu 2016). In recent decades, an increasing consensus has emerged that transporters and ion channels function as key components directly responsible for driving salt ion efflux and sequestration, thereby assisting plants in alleviating the toxicity of ion accumulation (Mickelbart et al. 2015). Vacuolar sequestration is a crucial mechanism for mitigating salt ion stress and has been the focus of extensive research on sodium (Ramakrishna et al. 2025). In contrast, studies on the transporters by which plants regulate the transport of chloride ions are still lagging behind those concerning sodium ions (Rajappa et al. 2024).

NRT is a class of nitrate transporter that generally includes the low‐affinity transporters (LATs) protein NRT1/PTR (NPF) and the high‐affinity transporters (HATs) NRT2 (Crawford and Glass 1998). Members of the NRT family play a crucial role in nitrate uptake during plant growth and are capable of transporting multiple substrates (Corratgé‐Faillie and Lacombe 2017; Wang et al. 2023). Interestingly, previous researchers have shown that certain NPF members possess both nitrate and chloride transport activities (Liu et al. 2020; Wen et al. 2017; Xiao et al. 2021). However, it has not been clearly elucidated how these dual‐substrate‐transporting NPFs exert their physiological functions in response to specific environmental stimuli. Moreover, a considerable proportion of NRT members were significantly upregulated in response to salt stress according to our previous research findings (Zhao et al. 2022). Although the transport of chloride by NRTs has been reported (Li et al. 2015; Wu et al. 2025), the mechanism by which NRTs transport chloride in plants requires further elucidation.

The expression of functional genes was up‐ or down‐regulated under various environmental conditions and signalling pathways, which are manipulated by different transcription factors (TFs) (Krasensky and Jonak 2012). Among the diverse categories of transcription factors, NAC TFs occupy a central role in the transcriptional regulation of plant abiotic stress responses (Han et al. 2023), encompassing plant responses to low temperature (Song et al. 2022), high temperature (Xi et al. 2022), drought (Sakuraba et al. 2020), and flooding (Christianson et al. 2009). Furthermore, salt stress is one of the critical abiotic stresses in which NAC transcription factors are involved in regulation. Overexpression of SINAC10 boosts *AtP5CS1/2* expression, thereby enhancing plant salt resistance (Du et al. 2022). BpNAC012 positively regulates salinity tolerance through an osmoregulation strategy (Hu et al. 2018). However, the mechanism by which NACs regulate ion homeostasis and sequestration is less well studied (Han et al. 2023).

Citrus stands as one of the largest fruit crops globally in terms of production and cultivation area (Li et al. 2025; Xu et al. 2013). However, the salt sensitivity of citrus is widely acknowledged in the agricultural community (Martínez‐Alcántara et al. 2015). Additionally, researchers have pointed out that the chloride ion, rather than the sodium ion, serves as the primary limiting factor for salt tolerance in citrus (Storey and Walker 1999; Moya et al. 2003; Brumós et al. 2009; Dahro et al. 2023). To provide novel insights into the mechanisms of salt response in citrus, we conducted an in‐depth study on *Citrus trifoliata* , a rootstock widely utilized in commercial production. Our study specifically targeted the *CtrNPF2.1* gene, selected based on physiological characterisation and transcriptome analysis. Through transgenic approaches and functional transport assays, we demonstrated that CtrNPF2.1 regulates chloride sequestration under salt stress and nitrogen utilisation under high‐nitrogen conditions by mediating the bidirectional transport of chloride and unidirectional transport of nitrate ions. Furthermore, we revealed that CtrNPF2.1 can form a dimeric structure and may facilitate chloride transport through the interfacial pore, providing novel insights into the functional significance of dimerisation in NRT family proteins. Additionally, we identified a transcription factor, CtrNAC019, that enhances the transcriptional expression of *CtrNPF2.1*. Collectively, our findings elucidate the dual functional role of the CtrNAC019‐CtrNPF2.1 module in positively regulating both salt tolerance and nitrate utilisation in plants.

## 2 Results

### 2.1 *CtrNPF2.1* Is Transcriptionally Upregulated by Chloride Salt Stress

**FIGURE 1:**
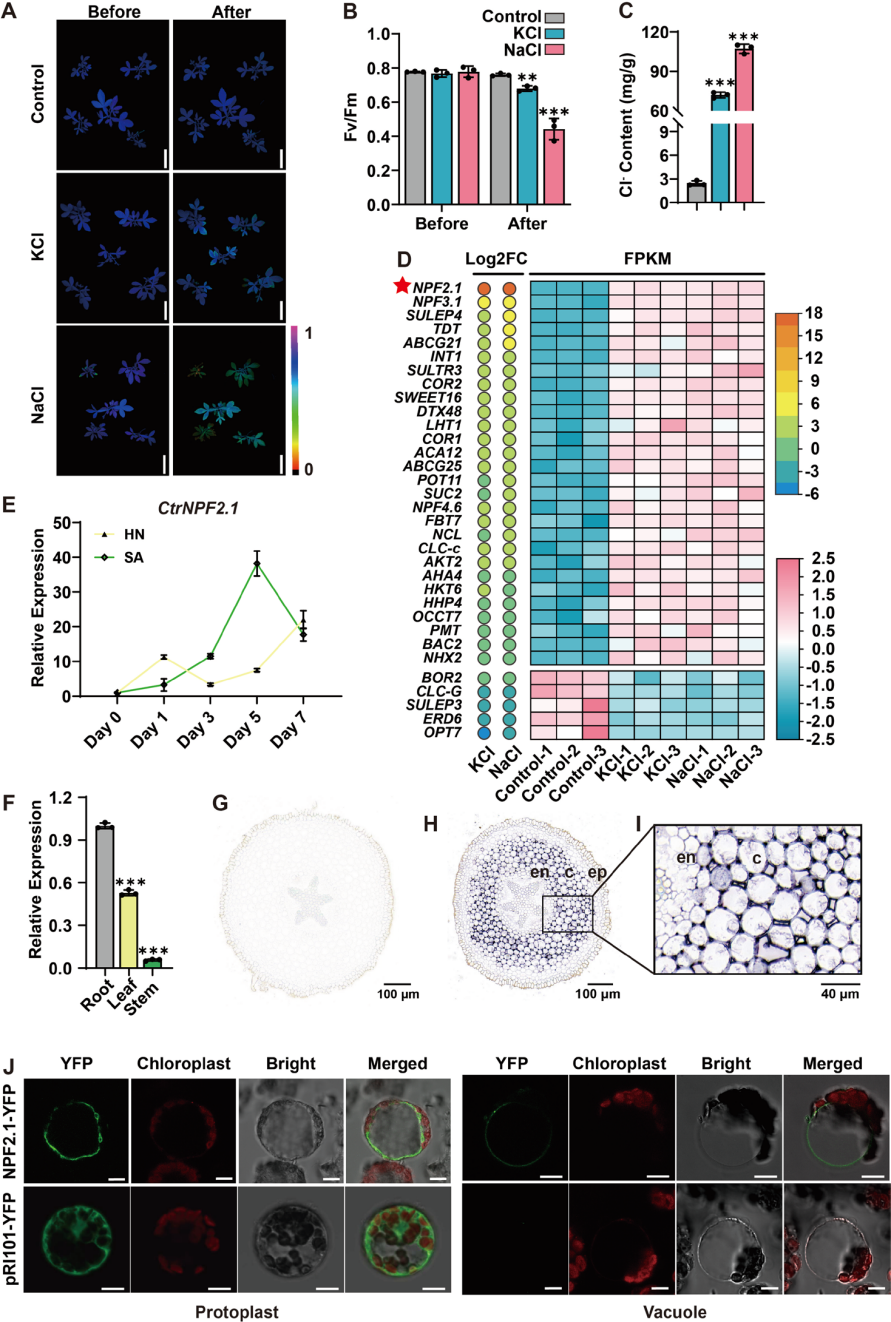
*CtrNPF2.1* was upregulated by high nitrate and chloride salt stress and had relatively high expression in the root cortex. (A, B) Chlorophyll fluorescence imaging (A) and Fv/Fm (Variable Fluorescence/Maximal Fluorescence) ratios (B) of trifoliate orange seedlings before and after treatments with 150 mM KCl, 150 mM NaCl and normal conditions (Control). Scale bars = 5 cm. (C) Chloride content determination following chloride salt stress treatment. (D) Expression profile of transporters from the set of 601 genes. Log2FC: Log fold change. FPKM: Fragments Per Kilobase of exon model per Million mapped fragments. (E) Transcription levels of *CtrNPF2.1* in samples treated with 10‐fold higher nitrate (HN: 50 mM nitrate) and 200 mM NaCl (SA). (F) Transcription levels of *CtrNPF2.1* in root, leaf, and stem tissues. Values represent the mean ± SE with three biological replicates. The asterisks indicate significant differences as assessed by independent samples *t*‐test, **0.01 > *p* > 0.001, ***p < 0.001. (G–I) In situ hybridisation assay. Negative control (G) and *CtrNPF2.1* (H) in situ hybridisation on cross sections of trifoliate orange roots (10 mm from root tip). (I) A partially enlarged image of the region marked by the black box in (H). ep, epidermis; c, cortex; en, endodermis. (J) Confocal microscopy images showing isolated mesophyll protoplasts (left) and released vacuoles (right) from tobacco leaves expressing CtrNPF2.1 fused with YFP, as well as those transformed with the empty vector control fluorescent protein. Scale bars = 10 μm.

To investigate the mechanisms by which trifoliate orange (*Citrus trifoliata L*.) mitigates chloride toxicity, trifoliate orange seedlings were subjected to high concentrations (150 mM) of two forms of chloride salt: sodium chloride (NaCl) and potassium chloride (KCl). There was a remarkable reduction in chlorophyll fluorescence intensity in the treated plants compared with the control samples (Figure 1A,B), indicating that chloride salts caused stress‐related damage in trifoliate orange. In both treated groups, the concentration of chloride ion (Cl^−^) exhibited a significant increase (Figure 1C). However, the concentrations of sodium (Na^+^) and potassium (K^+^) ions increased only in their respective corresponding samples (Figure S1). We employed parallel treatments of NaCl and KCl combined with transcriptome intersection analysis, aiming to identify chloride‐specific response genes at the transcriptional level and effectively eliminate the interference of cations. Given that the root system serves as the primary tissue responsible for ion absorption and transport to the shoot, root samples from 7‐day salt‐treated trifoliate orange plants and corresponding control samples were collected for transcriptome sequencing analysis. Comparative analysis identified 836 differentially expressed genes (DEGs) following potassium chloride treatment and 1234 DEGs following sodium chloride treatment across all time points relative to the control group (File S1). The Venn diagram illustrates that there are 601 overlapping genes between the two treatment groups (Figure S2A). We hypothesized that these 601 genes are specifically upregulated in response to chloride salt stress. Notably, these 601 genes exhibited significant enrichment in transcription factors and transporter‐related pathways (Figure S2B,C), highlighting the pivotal role of transcriptional regulation and transport mechanism in the trifoliate orange's adaptation to chloride stress. Importantly, by analysing the expression pattern of transporter members, *CtrNPF2.1* was identified as a promising candidate gene that is significantly induced under both chloride salt stress conditions for further investigation (Figure 1D).

### 2.2 CtrNPF2.1 is Primarily Expressed in Root Cortex and Localized to Tonoplast

According to our previous report (Zhao et al. 2022), CtrNPF2.1 is a member of the low‐affinity nitrate transporter NRT1/PTR subfamily and exhibits the highest sequence similarity to AtNPF2.9 (also known as AtNRT1.9) in *Arabidopsis thaliana* . To characterise the transcriptional pattern of *CtrNPF2.1*, we evaluated its expression levels under high nitrate (HN), low nitrate (LN), and salt stress (SA) conditions, as well as across various tissues. The results demonstrated that the expression levels of *CtrNPF2.1* were induced under conditions of high nitrate and salt stress (Figure 1E), but there was no induction or inhibition in response to the low nitrate environment (Figure S3). Furthermore, it is important to highlight that *CtrNPF2.1* had a relatively higher expression level in the root (Figure 1F), implying a potential role of CtrNPF2.1 in ion or nitrogen transport in the root system. To elucidate the tissue‐specific distribution of CtrNPF2.1, an in situ hybridisation staining assay was conducted. The results indicated that CtrNPF2.1 is predominantly enriched in root cortex cells (Figure 1G–I). Laser confocal microscopy was employed to reveal that the CtrNPF2.1‐YFP signal was co‐localized with the tonoplast marker protein (Figure S4). We further confirmed the vacuole membrane localisation signal of CtrNPF2.1‐YFP by isolated protoplast and released vacuoles, with the empty vector serving as a control (Figure 1J). Collectively, these findings indicate that the tonoplast‐localized CtrNPF2.1 is predominantly expressed in root cortex cells and is upregulated under high nitrate and salt stress conditions. This evidence encourages further investigation into the role of CtrNPF2.1 in enhancing plant salt stress tolerance and nitrogen use efficiency.

### 2.3 CtrNPF2.1 Positively Enhances Salt Resistance and Promotes Root Growth

**FIGURE 2:**
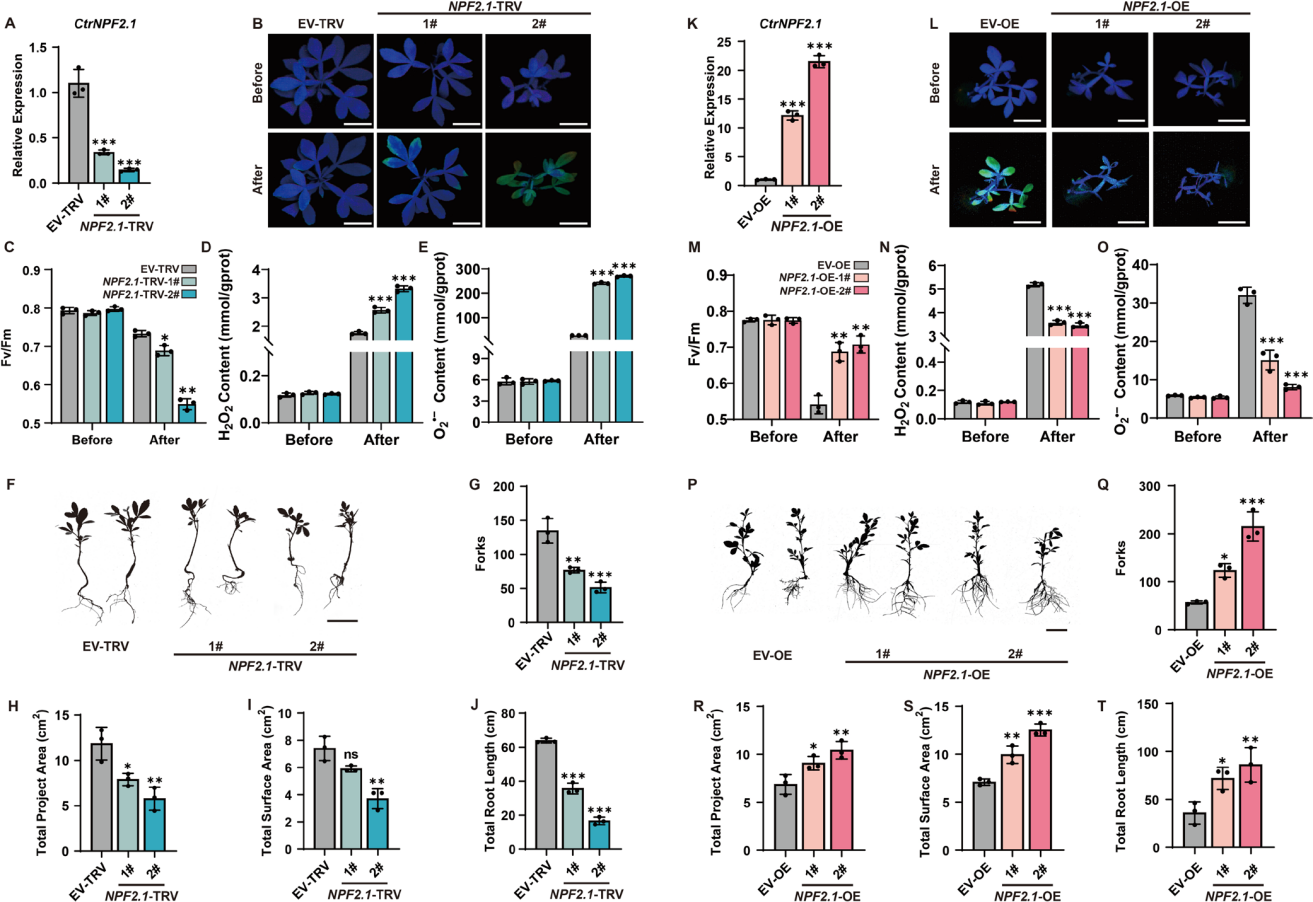
*CtrNPF2.1* positively regulate salt tolerance and root growth in trifoliate orange. (A) The transcription level of *CtrNPF2.1* in EV‐TRV, *CtrNPF2.1*‐TRV‐1 and *CtrNPF2.1*‐ TRV‐2 plants. (B, C) Chlorophyll fluorescence imaging (B) and Fv/fm (C) ratios before and after 150 mM NaCl treatment. Scale bars = 1 cm. (D, E) Accumulation of in situ H_2_O_2_ (D) and O_2_^•‐^ (E) content before and after 150 mM NaCl treatment in EV‐TRV, *CtrNPF2.1*‐TRV‐1 and ‐2 plants. (F–J) Root scan imaging (F) and root formation parameters including number of forks (G), total project area (H), total surface area (I) and total root length (J) in EV‐TRV, *CtrNPF2.1*‐TRV‐1, *CtrNPF2.1*‐TRV‐2 plants under normal growth condition. Scale bars = 5 cm. (K) The transcription level of *CtrNPF2.1* in EV‐OE, *CtrNPF2.1*‐OE‐1 and *CtrNPF2.1*‐OE‐2 plants. (L, M) Chlorophyll fluorescence imaging (L) and Fv/fm ratios (M) before and after 200 mM NaCl treatment. Scale bars = 1 cm. (N, O) Accumulation of in situ H_2_O_2_ (N) and O_2_^•‐^ content (O) before and after 200 mM NaCl treatment in EV‐OE, *CtrNPF2.1*‐OE‐1 and *CtrNPF2.1*‐OE‐2 plants. (P–T) Root scan imaging (P) and root formation parameters including number of forks (Q), total project area (R), total surface area (S) and total root length (T) in EV‐OE, *CtrNPF2.1*‐OE‐1 and *CtrNPF2.1*‐OE‐2 plants. Scale bars = 5 cm. Values represent the mean ± SE with three biological replicates. The asterisks indicate significant differences as assessed by independent samples *t*‐test, ns, *p* > 0.05, *0.05 > *p* > 0.01, **0.01 > *p* > 0.001, ***p < 0.001.

In order to investigate the physiological function of CtrNPF2.1, transgenic plants with altered expression of *CtrNPF2.1* were generated using *Agrobacterium*‐mediated transformation. Specifically, the virus‐induced gene silencing (VIGS) method was employed to reduce the expression level of *CtrNPF2.1* in trifoliate orange. Two randomly selected groups from the transgenic seedlings were designated as group 1 (*CtrNPF2.1*‐TRV‐1#) and group 2 (*CtrNPF2.1*‐TRV‐2#), along with an empty vector (EV‐TRV) control group. The transcription level of *CtrNPF2.1* in the VIGS plants was significantly repressed, reaching approximately 30% and 10% of the control level in the two interference groups, respectively (Figure 2A). In the presence of salt treatment, the VIGS plants showed obvious salt sensitivity, as reflected by lower chlorophyll fluorescence compared to control plants (Figure 2B,C). Accordingly, the VIGS groups accumulated more reactive oxygen species (ROS), including H_2_O_2_ and O_2_^•‐^, than the control plants under salt stress (Figure 2D,E), indicating that interference with *CtrNPF2.1* expression substantially compromises the salt tolerance of trifoliate orange. In addition, under the same growth conditions, VIGS plants displayed a phenotype marked by compromised root development (Figure 2F), exhibiting significant reductions in root length, surface area, and branching frequency compared to the control group (Figure 2G–J). *Agrobacterium*‐mediated root‐hair transformation was applied to achieve overexpression of *CtrNPF2.1* in trifoliate orange (Figure S5A). The overexpression efficiency was greater than 10‐fold for group 1 (*CtrNPF2.1*‐OE‐1#) and 20‐fold for group 2 (*CtrNPF2.1*‐OE‐2#) compared with the empty vector (EV‐OE) control (Figure 2K). Under salt stress, the overexpression group plants exhibited significantly higher Fv/fm ratios and lower ROS accumulation compared to the control group (Figure 2L–O), suggesting that increased expression levels of *CtrNPF2.1* could enhance the salt resistance of trifoliate orange. Besides, the root growth of the *CtrNPF2.1*‐OE plants demonstrated a remarkable enhancement in vigour compared to that of the EV group plants (Figure 2P–T). We further determined the expression level and enzyme activity of nitrate reductase (NR), which can reflect the efficiency of nitrogen assimilation and utilisation. It was observed that the expression level and enzyme activity of NR were significantly higher with the overexpression of *CtrNPF2.1* compared to the control plants. Conversely, the opposite phenomenon was detected when *CtrNPF2.1* was silenced (Figure S5C–F). Moreover, we ectopically expressed *CtrNPF2.1* in *Arabidopsis*. The physiological phenotypes revealed a conserved and positive role of CtrNPF2.1 in enhancing plant salt tolerance and promoting root development (Figure S6), which supports the notion that CtrNPF2.1 may possess broad applicability across different plant species. A plausible hypothesis is that CtrNPF2.1 may regulate chloride and nitrate transport, thereby contributing to the observed phenotypes.

### 2.4 CtrNPF2.1 Manipulates Cl^−^ and NO_3_ ^−^ to Regulate Salt Tolerance and Root Growth

**FIGURE 3:**
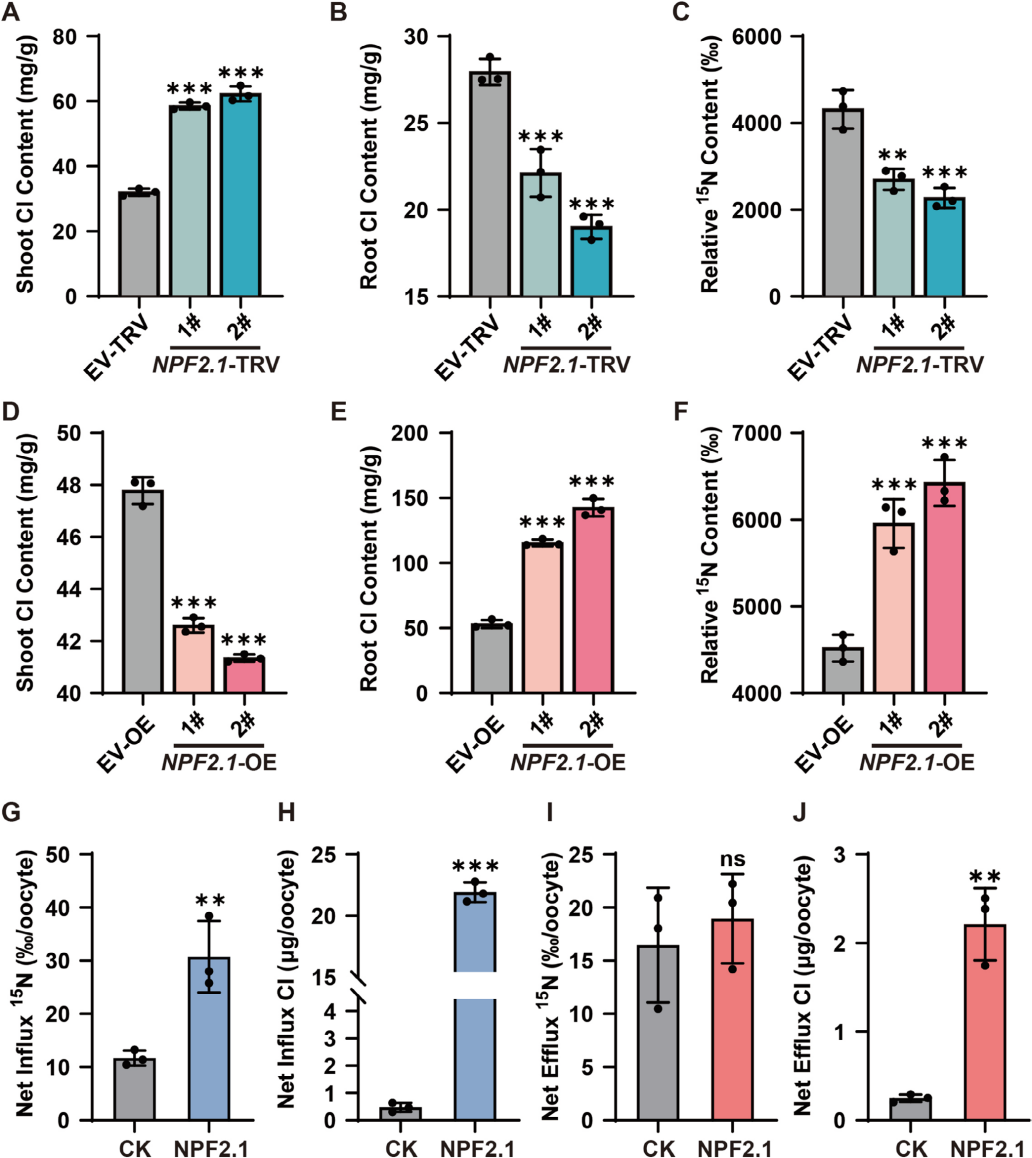
CtrNPF2.1 mediates chloride sequestration and nitrate efflux to influence plant salt tolerance and root growth. (A, B) Shoot (A) and root (B) chloride content in EV‐TRV, *CtrNPF2.1*‐TRV‐1, *CtrNPF2.1*‐TRV‐2 plants after 150 mM NaCl treatment. (C) Relative ^15^N content in EV‐TRV, *CtrNPF2.1*‐TRV‐1, *CtrNPF2.1*‐TRV‐2 plants root after 10 mM K^15^NO_3_ treatment. (D, E) Shoot (D) and root (E) chloride content in EV‐OE, *CtrNPF2.1*‐OE‐1, *CtrNPF2.1*‐OE‐2 plants after 200 mM NaCl treatment. (F) Relative ^15^N content in EV‐OE, *CtrNPF2.1*‐OE‐1, *CtrNPF2.1*‐OE‐2 plants root after 10 mM K^15^NO_3_ treatment. Values represent the mean ± SE with three biological replicates. (G–H) The absorption relative ^15^N and chloride content assay. Relative ^15^N (G) and chloride (H) of oocyte cells were detected after incubating with MBS + 50 mM KCl or MBS + 50 mM K^15^NO_3_. (I, J) The efflux relative ^15^N and chloride content assay. Each cell was injected 100 mM KCl or K^15^NO_3_. Relative ^15^N (I) and chloride (J) in oocyte cells were measured after incubating for 1 h in MBS. Each replicate contained 10 oocytes. Values represent the mean ± SE with three biological replicates. The asterisks indicate significant differences as assessed by independent samples *t*‐test, ns, *p* > 0.05, **0.01 > *p* > 0.001, ***p < 0.001.

To dissect the physiological function of CtrNPF2.1, the element content in transgenic materials was determined. The accumulation of chloride ions in the shoot tissues of *CtrNPF2.1* VIGS plants was significantly higher compared to that in control plants following salt stress treatment, while the chloride ion content in the roots of VIGS plants was markedly reduced (Figure 3A,B). This suggests that the suppression of *CtrNPF2.1* expression results in increased chloride ion accumulation in the shoot tissues. Meanwhile, a ^15^N labelled nitrate absorption assay was conducted. It was found that the uptake of ^15^NO_3_^−^ in the *CtrNPF2.1* VIGS group was markedly lower than that in the control group (Figure 3C), suggesting that silencing of *CtrNPF2.1* impairs the nitrogen uptake process. In contrast, more chloride accumulated in the roots, but not in the shoot tissues of *CtrNPF2.1* overexpressing plants (Figure 3D,E), along with increased isotope‐labelled nitrogen assimilation associated with higher expression of *CtrNPF2.1* (Figure 3F). Therefore, we speculate that CtrNPF2.1 may sequester chloride ions into the root and facilitate nitrate uptake in plants.

### 2.5 CtrNPF2.1 Functions as a Unidirectional NO_3_ ^−^ and Bidirectional cl^−^ Transporter

**FIGURE 4:**
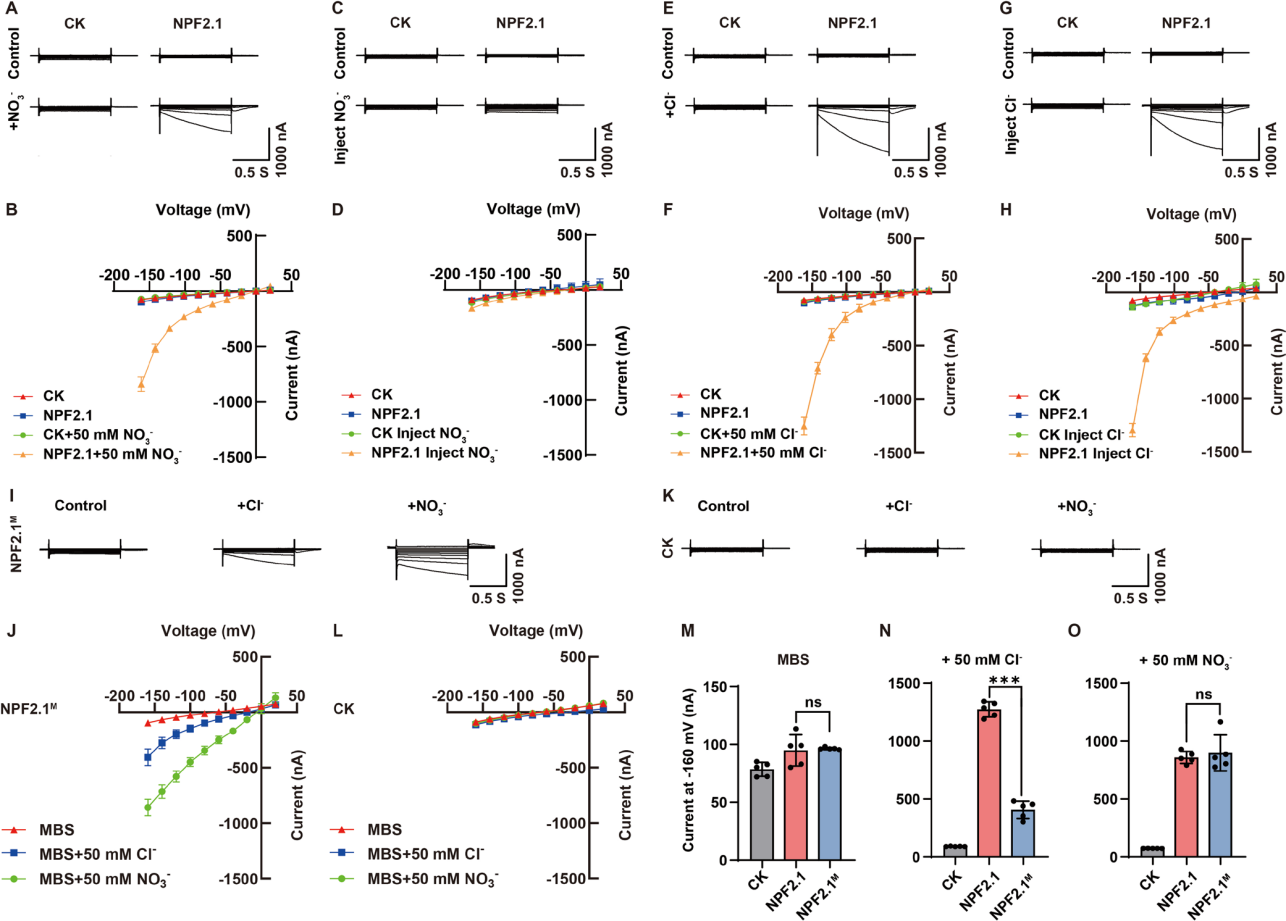
CtrNPF2.1 possesses dual‐transport activity for nitrate and chloride. (A–H) Examples of currents (A, C, E and G) and current–voltage (I–V) relationship (B, D, F and H) of *X. laevis* oocyte cells injected with water and *CtrNPF2.1* cRNA, *n* = 6. The bath solution concentrations of NO_3_^−^ (B) and Cl^−^ (F) were 50 mM and then the test current caused by substrate from apoplast to cytosol. 100 mM NO_3_^−^ (D) and Cl^−^ (H) was injected into oocyte cell and the test current caused by substrate from cytosol to apoplast. Examples of currents (A), (C), (E) and (G) correspond respectively to current–voltage relationship figures (B), (D), (F) and (H). MBS solutions had a pH of 5.5 and an osmolality of 210 mOsm. (I–L) Examples of currents (I, K) and current–voltage (I–V) relationship of *X. laevis* oocyte cells injected with TM3 and TM6 mutated *CtrNPF2.1* cRNA (J) and water (L), *n* = 5. Examples of currents (I) and (K) correspond respectively to current–voltage relationship figures (J) and (L). The TEVC experiments were repeated three times, with similar results. (M–O) The current values at −160 mV when water‐injected, *CtrNPF2.1* cRNA‐injected and mutated *CtrNPF2.1* cRNA‐injected oocyte cells incubated at MBS (M), MBS + 50 mM Cl^−^ (N) and MBS + 50 mM NO_3_^−^ (O), *n* = 5. Values represent the mean ± SE with indicated biological replicates. The asterisks indicate significant differences as assessed by independent samples *t*‐test, ns, *p* > 0.05, ***p < 0.001.

To further verify the transport activity and direction of CtrNPF2.1, we expressed *CtrNPF2.1* in *Xenopus laevis* oocytes and confirmed that the CtrNPF2.1 protein was successfully localized to the cell membrane of *X. laevis* oocytes (Figure S7). Then the uptake and efflux assays were conducted. The results showed that nitrate could only be taken up from the bath solution into the cytosol of oocyte cells expressing the CtrNPF2.1 protein (Figure 3G,I). Correspondingly, oocytes expressing CtrNPF2.1 absorbed and effluxed more chloride compared with water‐injected control cells (Figure 3H,J). Given the vacuole membrane CtrNPF2.1 protein was translocated and expressed in the plasma membrane of oocyte cells, the transport direction detected by CtrNPF2.1 expressed on the oocyte plasma membrane is opposite to that on the plant vacuole membrane (Figure S8). By utilizing the two‐electrode voltage clamp (TEVC) technology, dramatic voltage‐dependent currents were recorded upon exposure to 50 mM Cl^−^ or NO_3_^−^ at pH 5.5 in CtrNPF2.1‐expressing oocytes, but not in water‐injected control cells (CK), suggesting that CtrNPF2.1 possessed both transport activity for NO_3_^−^ and Cl^−^ (Figure 4A,B,E,F). Furthermore, oocytes expressing CtrNPF2.1 exhibited more activity at pH 5.5 compared to pH 7.5 when exposed to 50 mM Cl^−^ or NO_3_^−^ (Figure S9A,B). The increase in substrate concentration led to a more pronounced change in current (Figure S9C–F). Interestingly, we found that CtrNPF2.1‐expressing oocytes exhibited effective nitrate transport ability when nitrate substrate was added to the bath solution, but no significant difference in current in nitrate‐injected cells compared with CK cells (Figure 4A–D). In contrast, voltage‐clamp experiments with chloride as the substrate indicated activity on both sides (Figure 4E–H). These results demonstrate that CtrNPF2.1 is a bidirectional Cl^−^ and unidirectional NO_3_^−^ transporter that may modulate plant vacuolar chloride sequestration and nitrate efflux in response to chloride salt stress and high nitrate conditions.

Through protein crystal analysis, researchers have elucidated how the NRT protein captures nitrate via an active pocket and transports it across the membrane through conformational changes (Parker and Newstead 2014), but the chloride transport is still mysterious. The previous findings demonstrated that NRTs can form dimers through hydrophobic interactions between transmembrane regions TM3 and TM6, and the buried surface area between monomers is approximately 2136 Å^2^, which may have physiological significance (Parker and Newstead 2014; Sun et al. 2014). Considering that chloride transport by CtrNPF2.1 was not unidirectional (Figure 4E–H), we speculated that chloride ions might cross the membrane via the pore formed by TM3 and TM6 at the CtrNPF2.1 dimer interface. To test this hypothesis, we first conducted the bimolecular fluorescence complementation (BiFC) and luciferase complementation imaging (LCI) assays to demonstrate that CtrNPF2.1 could interact itself in vivo (Figure S10A,C). To further investigate the chloride transport mechanism of CtrNPF2.1, we identified all the potential residues that might interact with chloride ions through electrostatic interactions (arginine, Arg; lysine, Lys) or hydrogen bonding (serine, Ser; tyrosine, Tyr) (Figure S11A). All the presumptive residues around the transmembrane (TM) regions 3 and 6 were mutated to alanine (Ala, A), an electrically neutral residue. The mutant at the predicted TM3/TM6 interface of CtrNPF2.1 (NPF2.1^M^) was tested by TEVC and found the notably impaired chloride transport activity, but not nitrate transport activity or itself interaction (Figure 4I–O; Figure S10B,D). These imply that the residues at the dimeric interface might be involved in chloride ion transport mediated by CtrNPF2.1 (Figure S11B). However, the specific mechanism by which these amino acids regulate the chloride ion transport of CtrNPF2.1 requires further protein structural and functional investigation.

### 2.6 CtrNAC019 Binds to the Promoter of *CtrNPF2.1* and Activates Its Expression·

**FIGURE 5:**
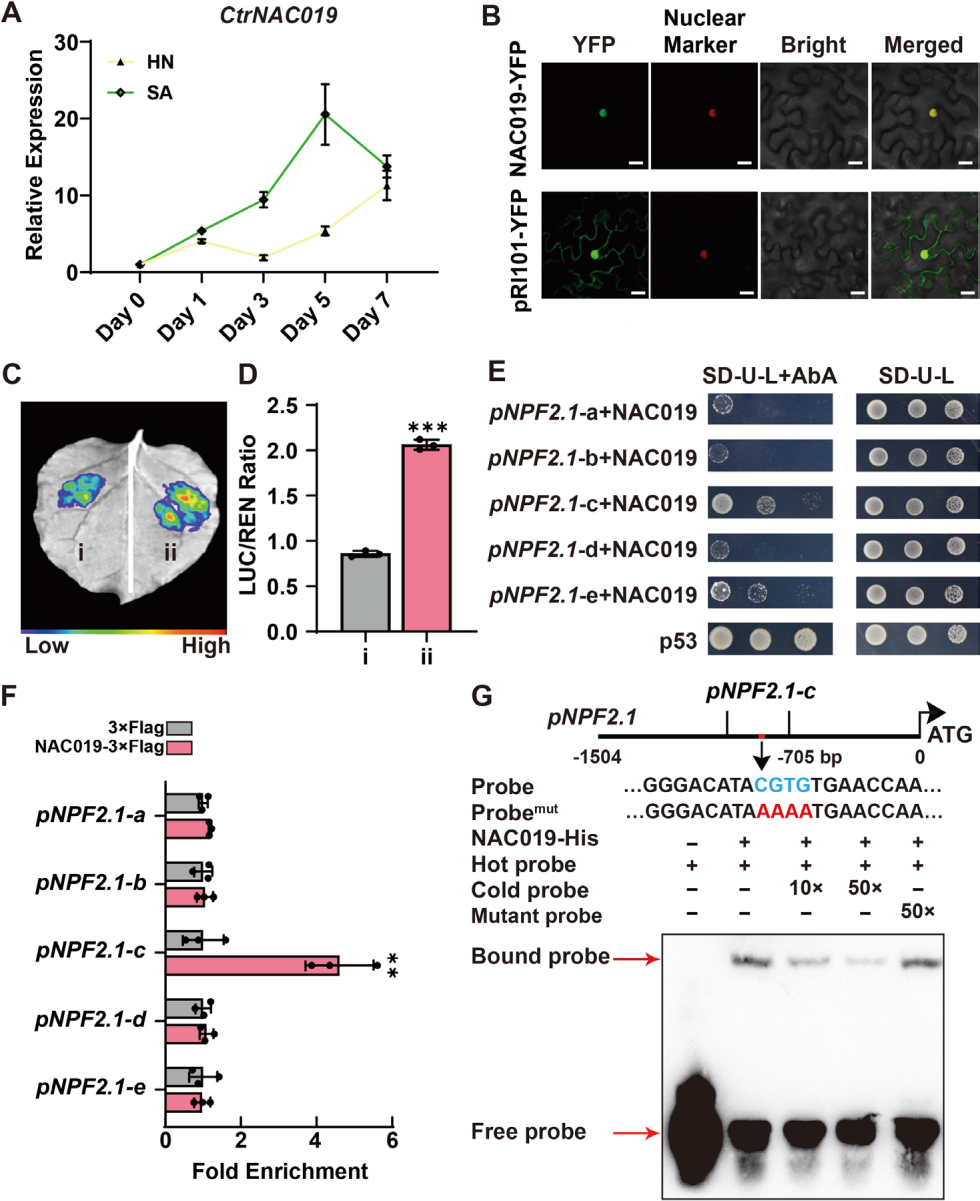
The expression of *CtrNPF2.1* is transcriptionally upregulated by CtrNAC019. (A) The expression levels of *CtrNAC019* under high nitrate (HN, 10‐fold NO_3_^−^) and salt stress (SA, 200 mM NaCl) conditions. (B) Subcellular location of CtrNAC019 tagged with YFP and empty vector (EV) in *N. benthamiana* leaves. Scale bars = 10 μm. (C, D) LUC imaging and luciferase activity assay. LUC bioluminescence imaging (C) and relative firefly luciferase activity assays (D) were performed in *N. benthamiana* leaves co‐transformed with SK‐CtrNAC019 and the pGreenII0800‐LUC reporter driven by promoter of *CtrNPF2.1* on the right side (ii). Injection of the empty vector on left side served as a control (i). Error bars indicate mean ± SE (*n* = 3). (E) Growth of yeast cells co‐transformed with different combinations of prey and baits on SD/‐Ura/−Leu medium added with 0 or 150 ng/mL aureobasidin A (AbA). CtrNAC019‐pGADT7 was used as a prey (NAC), while the promoter fragments a–e (Figure S12C) fused to pAbAi plasmid were used as baits (*pNPF2.1*‐a/b/c/d/e‐pAbAi). The pGADT7 and different baits were used as negative controls in Figure S12D, and p53‐AbAi + pGAD‐p53 was used as a positive control. The yeast with OD_600_ = 0.2 was diluted into 10^−1^, 10^−2^, 10^−3^. (F) ChIP‐qPCR assay. The amplified primers were designed according to Figure S12C. Chromatin immunoprecipitation was performed via using samples with stably expression of CtrNAC019‐Flag or empty Flag control. ChIP‐qPCR was conducted to assess the enrichment of fragment b, c, e containing NAC binding sites and a, d lacking binding site. The relative of enrichment in empty flag control sample was set as ‘1’. Values represent the mean ± SE with three biological replicates. The asterisks indicate significant differences as assessed by independent samples *t*‐test, ns, *p* > 0.05, *0.05 > *p* > 0.01, **0.01 > *p* > 0.001, ***p < 0.001. (G) EMSA analysis. Biotin‐free labelled probe was used as competitor (cold probe), and mutation from the CGTG to AAAA element caused the lose competition of cold probe.

To elucidate the molecular regulatory mechanism of CtrNPF2.1, we conducted a comprehensive screening of transcription factors from differentially expressed genes (DEGs) identified in our transcriptome data. This analysis led to the identification of 19 transcription factors as candidate genes (Figure S12A). The yeast one‐hybrid (Y1H) assay was performed to validate binding capabilities of target TFs to *CtrNPF2.1* promoter sequence (*pNPF2.1*). It is noteworthy that *CtrNAC019* exhibited the binding capacity to *CtrNPF2.1* promoter (Figure S12B). Similar salt‐ and high nitrate‐induced expression patterns of *CtrNAC019* compared with *CtrNPF2.1* were determined via RT‐qPCR (Figure 5A). Subcellular localisation study demonstrated that CtrNAC019 was specifically localized to the nucleus (Figure 5B). Dual luciferase complementation and luciferase enzyme activity assays confirmed that CtrNAC019 positively regulate the transcriptional activity of *CtrNPF2.1* promoter (Figure 5C,D). The promoter sequence of *CtrNPF2.1* was further divided into five fragments labelled a–e according to binding motifs distribution (Figure S12C). Y1H and chromatin immunoprecipitation (ChIP)‐qPCR further indicated that CtrNAC019 bind to c fragment of *CtrNPF2.1* promoter (Figure 5E,F; Figure S12D). The result of electrophoretic mobility shift assay (EMSA) suggested that a key motif of CGTG located −705 bp upstream of *CtrNPF2.1* coding sequence was bound by the CtrNAC019 (Figure 5G). In brief, we identified that CtrNAC019 binds to the promoter of *CtrNPF2.1* and positively regulates its transcription level in response to salt stress and high nitrate conditions.

### 2.7 CtrNAC019 Positively Modulates Salt Tolerance and Root Growth by Enhancing *CtrNPF2.1* Expression

**FIGURE 6:**
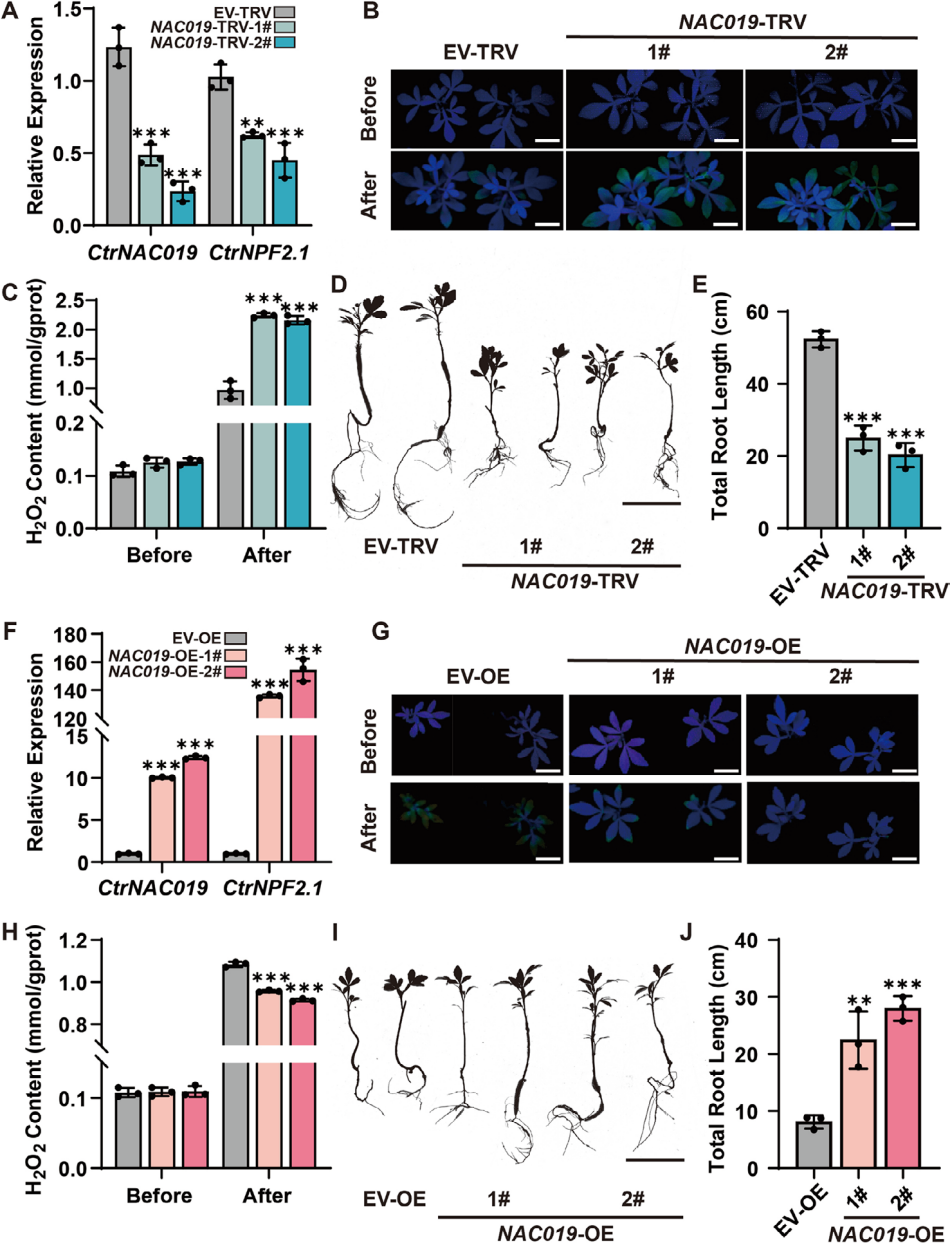
CtrNAC019 regulates the transcription level of *CtrNPF2.1* to modulate salt tolerance and root growth in trifoliate orange. (A) The transcription levels of *CtrNAC019* and *CtrNPF2.1* in EV‐TRV, *CtrNAC019*‐TRV‐1 and *CtrNAC019*‐TRV‐2 plants. (B) Chlorophyll fluorescence imaging before and after 150 mM NaCl treatment. Scale bars = 1 cm. (C) Accumulation of in situ H_2_O_2_ content in trifoliate orange before and after 150 mM NaCl treatment. (D, E) Root scan imaging (D) and total root length assay (E) of EV‐TRV and *CtrNAC019*‐TRV seedlings under normal condition. Scale bars = 10 cm. (F) The transcription levels of *CtrNAC019* and *CtrNPF2.1* in EV‐OE, *CtrNAC019*‐OE‐1# and *CtrNAC019*‐OE‐2# plants. (G) Chlorophyll fluorescence imaging before and after 200 mM NaCl treatment. Scale bars = 1 cm. (H) Accumulation of in situ H_2_O_2_ content in trifoliate oranges before and after 200 mM NaCl treatment. (I, J) Root scan imaging (I) and total root length assay (J) of EV‐OE and *CtrNAC019*‐OE seedlings under normal condition. Scale bars = 10 cm. Values represent the mean ± SE with three biological replicates. The asterisks indicate significant differences as assessed by independent samples *t*‐test, ** 0.01 > *p* > 0.001, ***p < 0.001.

To further elucidate the physiological function of CtrNAC019, VIGS‐induced plants with specifically silenced *CtrNAC019* and overexpressed transgenic plant materials were generated. Results of RT‐qPCR revealed that interference of *CtrNAC019* remarkably reduced the expression of *CtrNPF2.1*, no matter under control and high nitrate or salt stress conditions (Figure 6A; Figure S13A). Salt stress treatment suggested that *CtrNAC019*‐TRV plants exhibited attenuated Fv/fm ratios compared with EV‐TRV control (Figure 6B; Figure S13B). An increase in ROS content was also observed in the *CtrNAC019* VIGS groups (Figure 6C; Figure S13C). Additionally, there was a remarkable hindrance to root growth in *CtrNAC019*‐TRV plants compared with control plants (Figure 6D,E; Figure S13D–F). Besides, we transiently overexpressed *CtrNPF2.1* in the *CtrNAC019*‐TRV background. Gene expression and physiological phenotypes indicated that the overexpression of *CtrNPF2.1* could reverse the root growth inhibition and salt sensitivity phenotypes of *CtrNAC019*‐TRV plants (Figure S14), genetically demonstrating that *CtrNPF2.1* is a downstream target gene of CtrNAC019. By contrast, an enhancement in the transcription level of *CtrNPF2.1* was observed in *CtrNAC019* overexpression plants (Figure S5B, Figure 6F). Noteworthily, plants of the *CtrNAC019*‐OE group exhibited an altered phenotype characterized by improved salt resistance and root growth (Figure 6G,I). Subsequently, the assays of ROS content and root parameter inspection confirmed this tendency (Figure 6H,J; Figure S15). These experiment results provided evidence that CtrNAC019 could regulate trifoliate orange salt tolerance and root development by modulating the transcription level of *CtrNPF2.1*.

### 2.8 CtrNAC019‐CtrNPF2.1 Module Regulates Salt‐Enhanced Nitrate Utilisation (SENU) and Nitrate‐Mediated Salt Tolerance (NMST)

**FIGURE 7:**
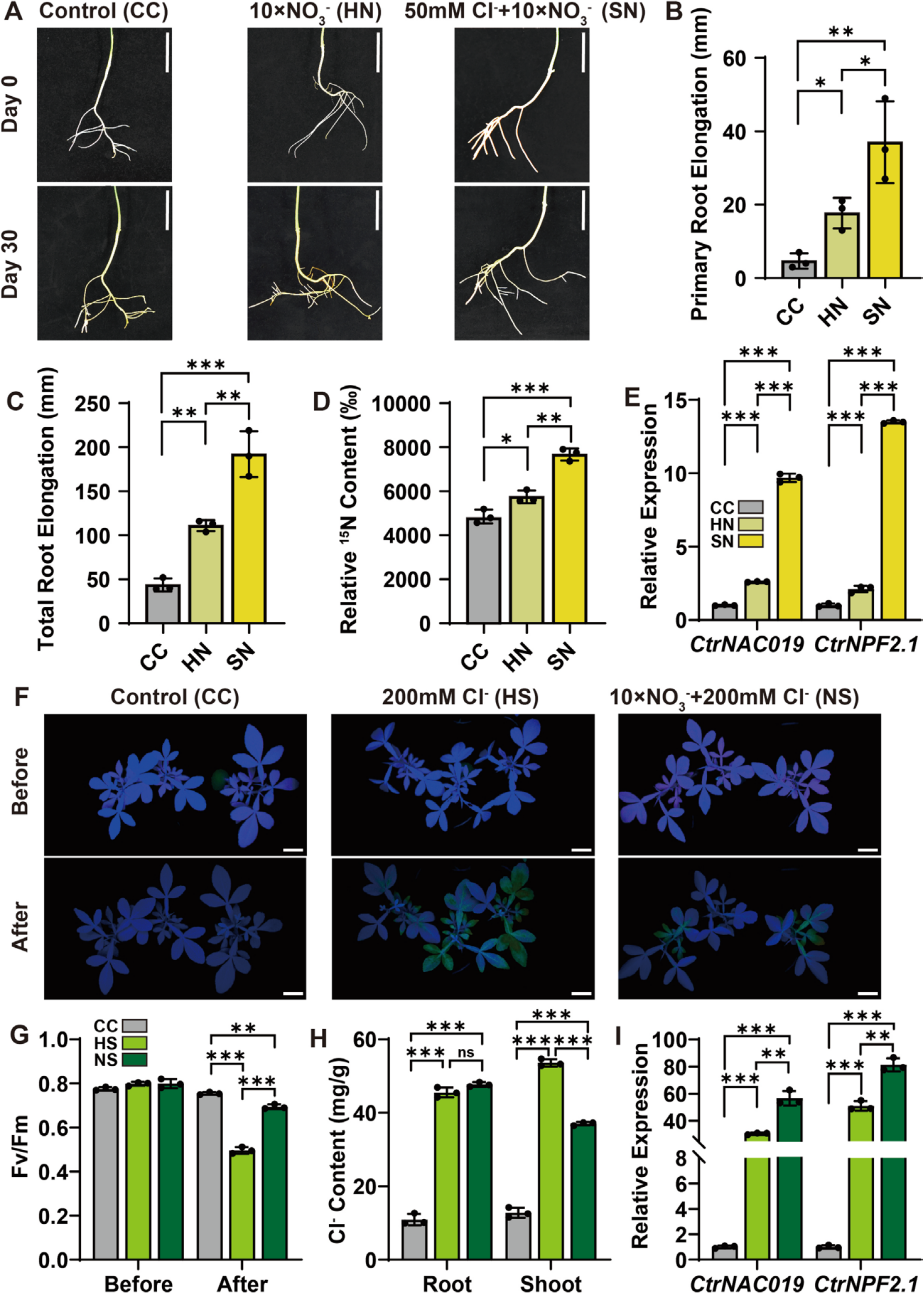
Moderate salt improves the nitrate mediated root growth and relative high nitrate enhances the salt tolerance in trifoliate orange. (A) Root photograph of 0 Day and 30th Day during corresponding treatment. CC, normal control conditions. HN, 10‐fold nitrate (50 mM NO_3_^−^) nutrition solution. SN, 50 mM NaCl pretreatment for 7 days and then transformed to 10‐fold nitrate nutrition solution. Scale bars = 2 cm. (B) The changed value of primary root length (subtract 0 Day from 30th Day). (C) The sum of changed value of all the root length (subtract 0 Day from 30th Day). (D) Relative ^15^N content of CC, HN, SN after 10 mM K^15^NO_3_ treatment for 10 h, respectively. (E) Transcription levels of *CtrNAC019* and *CtrNPF2.1* in CC, HN, SN plants. Values represent the mean ± SE with three biological replicates. (F‐G) Chlorophyll fluorescence imaging (F) and Fv/Fm ratios (G) before and after corresponding treatment. CC, normal control conditions. HS, 200 mM NaCl with nutrition solution. NS, 50 mM KNO_3_ pretreatment for 3 days and then transformed to 200 mM NaCl with nutrition solution. Scale bars = 1 cm. (H) The chloride content assay in root and shoot. (I) Transcription levels of *CtrNAC019* and *CtrNPF2.1* in CC, HS, NS plants. Values represent the mean ± SE with three biological replicates. The asterisks indicate significant differences as assessed by independent samples t‐test, ns, *p* > 0.05, *0.05 > *p* > 0.01, **0.01 > *p* > 0.001, ***p < 0.001.

The analysis of the dual functions of CtrNPF2.1 and the dual response pathways to high nitrate and salt stress provided insight into the potential regulatory mechanisms linking nitrogen and salt stress pathways. To further investigate whether the CtrNAC019‐CtrNPF2.1 module can respond to salt or high nitrate signalling and cross‐regulate root growth and salt resistance, trifoliate seedlings were subjected to the following treatments: (1) normal control conditions (CC); (2) relative high nitrate conditions (HN); (3) gentle NaCl pretreatment (50 mM NaCl) followed by relatively high nitrate conditions (SN). It was clearly depicted from Figure 7A–C that root growth activity was remarkably improved in SN compared to HN and CC. Notably, a simultaneous ^15^N‐labelled nitrate absorption experiment reflected that nitrate assimilation was significantly higher in plants from the SN group compared to the HN and CC groups (Figure 7D). Additionally, slight salt and high nitrate treatment enhanced the transcription levels of *CtrNPF2.1* and *CtrNAC019* according to expression analysis (Figure 7E). Conversely, we treated trifoliate seedlings to control conditions with high nitrate solution followed by salt stress (CC: normal conditions at all time, HS: salt treatment, NS: nitrate and salt treatment). Interestingly, compared to the HS group without nitrate pretreatment, the NS group exhibited a significantly enhanced salt tolerance phenotype (Figure 7F,G). Subsequently, chloride content analysis revealed that chloride accumulation in shoot was lower in the NS group compared to the HS group. Whereas, no significant difference was observed in chloride accumulation in root (Figure 7H). Importantly, quantification expression assay demonstrated that the transcription levels of *CtrNPF2.1* and *CtrNAC019* were significantly upregulated in the NS group plants compared to those in the CC and HS groups (Figure 7I). In addition, to address the CtrNAC019‐CtrNPF2.1 module's indispensable role, we have repeated the SENU and NMST experiments using *CtrNPF2.1*‐silenced (TRV) plants to functionally validate the module's involvement. The results demonstrated that the phenotypes of SENU and NMST were impaired to a certain extent when compared with those of the wild type plants (Figure 7; Figure S16), which provide direct functional evidence that CtrNPF2.1 is required for SENU and NMST. A brief summary can be illustrated that the CtrNAC019‐CtrNPF2.1 module participated in the gentle salt or high nitrate response for manipulating nitrate‐utilisation‐induced root morphogenesis and alleviating salt‐induced chloride accumulation in shoot.

## 3 Discussion

Salt stress and the excessive application of nitrogen fertilizers pose significant challenges to terrestrial crops today (Liu et al. 2022; Zhou et al. 2023). Genetic improvement aimed at developing salt‐tolerant and nitrogen‐efficient cultivars is crucial for maintaining stable food production and substantially contributing to sustainable food security. However, an effective module for enhancing both plant salt resistance and nitrogen utilisation has yet to be proposed. To this end, proposing a novel mechanism by which plant underground tissues simultaneously improve salt tolerance and nitrogen utilisation holds important practical implications. Herein, we report that CtrNAC019 triggers the expression of *CtrNPF2.1*, which orchestrates vacuolar chloride sequestration and nitrate efflux to enhance plant salt resistance and nitrogen utilisation.

Previous studies have demonstrated that transcriptional regulation and transport of salt ions are important processes involved in the formation of salt tolerance in plants (Dai et al. 2018). To search for key factors in citrus mitigation of chlorotoxin, we implemented NaCl and KCl treatments on trifoliate seedlings and sequenced the transcriptome. In the present work, enrichment analysis of the transcriptome also revealed that the two salt‐differentiated genes were dramatically enriched in transcriptional regulation and transporter activity pathways. A member of the nitrate transporter protein family, *CtrNPF2.1*, was identified to be induced by both chlorinated salts (NaCl and KCl). We further verified that CtrNPF2.1 was induced by high nitrate conditions but not low nitrate (Figure 1). Here, we discovered that CtrNPF2.1 was a new tonoplast‐localized member to mediate nitrogen utilisation and root growth in *Citrus trifoliata* based on plant growth and nitrogen content assays (Figures 1, 2, 3). It is worth mentioning that the vacuole not only serves as storage depots for nitrate, but also for salt ion sequestration under salinity stress. Both CLCc and ALMT9 have been suggested to function as vacuole chloride ion sequestration for plant salt tolerance (Baetz et al. 2016; Wu and Li 2019). However, there are still no studies reporting that root‐specific tonoplast transporter mediates salt‐stressed chloride sequestration in company with nitrogen utilisation.

The physiological and phenotypic evidence from transgenic materials indicates that *CtrNPF2.1* positively regulates the plant's capacity to withstand chloride salinity stress (Figure 3). CtrNPF2.1 was proven to be a vacuole membrane‐localized protein induced by chloride salts and to possess chloride ion transport activity (Figure 3H,J), suggesting that it may assist plants in sequestering chloride ion into vacuoles and limiting their leakage into the cytoplasm under salt stress conditions. The phenotype of the transgenic plants also supports this view, that overexpression of *CtrNPF2.1* in the root system leads to more chloride being enriched in the roots rather than in shoots (Figure 3D,E). Besides, we unexpectedly discovered that the root growth of *CtrNPF2.1* transgenic plants differed significantly from that of the empty vector control. Additionally, the ^15^N absorption assay in trifoliate orange plants indicated that CtrNPF2.1 positively affects the nitrate uptake ability (Figure 3). The oocytes with the expression of CtrNPF2.1 displayed an enhanced capacity for transporting nitrate into the cytoplasmic space, while there was no significant alteration in the transport in the opposite direction when compared to the control group (Figure 3G,I), indicating that CtrNPF2.1 may facilitate nitrate export from plant root vacuoles into the cytoplasm. Given the vacuolar membrane localisation characteristic of the CtrNPF2.1 protein, we hypothesise that the decreased exogenous ^15^N uptake and accumulation in *CtrNPF2.1*‐silenced plants might be an indirect result of the disruption of vacuolar nitrate homeostasis (He et al. 2017; Shi et al. 2023). Vacuoles serve as crucial storage compartments in plant cells. When the function of CtrNPF2.1 is constrained, it may cause nitrogen to accumulate in the vacuoles, which cannot be efficiently transported to the cytoplasm for assimilation and utilisation, thus creating the illusion of ample nitrogen storage. To support this hypothesis, we indicated that *CtrNPF2.1* silencing significantly diminished both NR transcript levels and enzyme activity, whereas overexpression enhanced them (Figure S5C–F). These changes are consistent with the alterations in ^15^N accumulation, suggesting that CtrNPF2.1‐mediated vacuolar nitrate homeostasis affects nitrate uptake and assimilation. To further explore the potential application value of CtrNPF2.1, we introduced *CtrNPF2.1* into *Arabidopsis* and observed that CtrNPF2.1 positively regulated salt tolerance and nitrogen utilisation efficiency (Figure S6). These findings imply that CtrNPF2.1 may have a broad‐spectrum effect on salt resistance and nitrate‐mediated root development in different plant species.

The NRT family members play a crucial role for plant nitrate transport and utilisation. Moreover, multiple studies have demonstrated that the plasma membrane members of the *NPF* family possess dual transport activities for nitrate and chloride (Wen et al. 2017; Wu et al. 2025; Xiao et al. 2021). But the chloride and nitrate dual transporter of NPF on the vacuole membrane has few reported so far. In this study, we identified CtrNPF2.1 shared the similar transport activity of nitrate, but a novel transport activity for chloride based on TEVC analysis (Figure 4). The electrophysiology assay of different pH value also revealed that CtrNPF2.1 might be a proton dependent transporter (Figure S9). Furthermore, we noticed that the transport activity of CtrNPF2.1 was also mediated by substrate concentrations, which was consistent with the previous reports (Wu et al. 2025; Xiao et al. 2021). In addition, we found that CtrNPF2.1 can interact with itself and may mediate the transport of chloride ions along the concentration gradient through the pore formed by the interaction interface based on site mutation assays (Figure 4I–O). The pore of interface with enigmatic physiological significance was reported with by previous NRT structure analysis (Parker and Newstead 2014; Sun et al. 2014). As research deepened, researchers reported the chloride ion transport activity of NRT members, but none clearly revealed the relationship between the dimeric pore and chloride ion transport (Li et al. 2015; Wen et al. 2017; Wu et al. 2025; Xiao et al. 2021). Herein, the site mutation of CtrNPF2.1^M^ with key residues in transmembrane domain 3/6 (TM3/6) binding interface decreased chloride transport activity, while no hindrance in self‐interaction (Figure S10B,D). Therefore, we propose that the pore formed by the TM3/6 of the CtrNPF2.1 dimeric binding interface might mediate the chloride ion transport function of CtrNPF2.1. Whether the identified residues at the binding interface contribute to a chloride permeation pore, constitute part of a chloride recognition site, or mediate the conformational changes necessary for chloride transport requires further structural and functional investigation.

Although both *CtrNPF2.1* and *AtNPF2.9* are members of the *NPF2* subfamily and possess high sequence similarity, their functional characteristics vary significantly. AtNPF2.9 is localized to the plasma membrane of root phloem cells and involved in phloem nitrate transport, which affects nitrate allocation to roots (Wang and Tsay 2011). Conversely, CtrNPF2.1 is localized to the tonoplast of root cortical cells and demonstrates dual‐transport activity for nitrate and chloride. This functional divergence might be a reflection of species‐specific adaptations: *Arabidopsis*, an annual herb, gives priority to nitrate distribution through the phloem, whereas citrus, a perennial woody plant, may place emphasis on vacuolar storage and remobilisation in roots to deal with fluctuating nitrogen availability and chloride salinity stress. The chloride‐transport capacity of CtrNPF2.1, which is lacking in AtNPF2.9, further implies neofunctionalisation in citrus as an adaptation to chloride‐stress environments. These comparisons underscore the evolutionary plasticity within the NPF family and offer a context for comprehending CtrNPF2.1's dual role in salt tolerance and nitrogen use efficiency.

To elucidate the transcriptional regulation of *CtrNPF2.1* in response to salt and high nitrogen conditions, CtrNAC019 was identified to bind to the promoter of *CtrNPF2.1* based on transcriptome data and Y1H assay (Figure 5; Figure S12). ChIP‐qPCR and EMSA assays (Figure 5F,G) further support the conservatism of the binding site compared with a previous study (Han et al. 2023). Correspondingly, we figured out that CtrNAC019 positively modulated citrus salt tolerance and root growth by enhancing *CtrNPF2.1* expression according to the transgenic plant phenotypes (Figure 6). NAC was reported to be involved in various biological processes including the development of the root and the regulation of salt tolerance (Takasaki et al. 2010; Xie et al. 2023). In this study, we filled the gap in understanding the involvement of NAC transcription factors in regulating the expression level of transporter genes that maintain ionic homeostasis and enable ion sequestration (Han et al. 2023). We innovatively elucidated the dual role of the CtrNAC019‐CtrNPF2.1 module in response to sufficient nitrate and chloride salt (Figure 1E and Figure 5A). Given the correspondence to both salt stress and high nitrogen, the upstream regulation mechanisms of the CtrNAC019‐CtrNPF2.1 module mediated by these two separate signalling pathways need to be further studied.

Chloride and nitrate ions are monovalent anions that serve as crucial sources of elemental chlorine and nitrogen for plant nutrition (Li, Qiu, et al. 2017; Li, Tester, and Gilliham 2017). Appropriate concentrations of chlorine are essential mineral elements for the growth and development of higher plants (Geilfus 2018). It has also been demonstrated that Cl^−^ can facilitate the absorption and utilisation efficiency of NO_3_^−^ (Lucas et al. 2024; Rosales et al. 2020). Interestingly, nitrate also exerted a potential influence on chloride ion uptake in plants subjected to salt stress. Different levels of nitrate availability alleviated salt stress injury in mustard plants by modulating proline and ethylene signalling pathways (Iqbal et al. 2015). The previous report indicated that nitrate‐responsive OsMADS27 may enhance salt tolerance in rice through the regulation of ion homeostasis and scavenging of reactive oxygen species (Alfatih et al. 2023). Appropriate concentrations of chlorine are essential mineral elements for the growth and development of higher plants (Geilfus 2018). However, the key factors and molecular mechanisms regarding how nitrate and chloride ions influence each other remain unclear. In this study, we experimentally verified the interactive effects of nitrogen and chlorine concurrently based on the dual‐reverse transporter CtrNPF2.1 of chloride ions and nitrate (Figures 3 and 4). We demonstrated that a low level of chlorine application enhances the plant's nitrogen use efficiency, while nitrogen pretreatment positively regulates the plant's salt tolerance, as characterized by the plant growth phenotypes (Figure 7A–CF,G). The alterations in the transcript levels of *CtrNAC019* and *CtrNPF2.1* were meticulously investigated according to the results of elemental chlorine and ^15^N content measurements (Figure 7D,E,H,I). These results support the dual‐function of CtrNAC019‐CtrNPF2.1 module in salt tolerance and nitrogen use efficiency.

**FIGURE 8:**
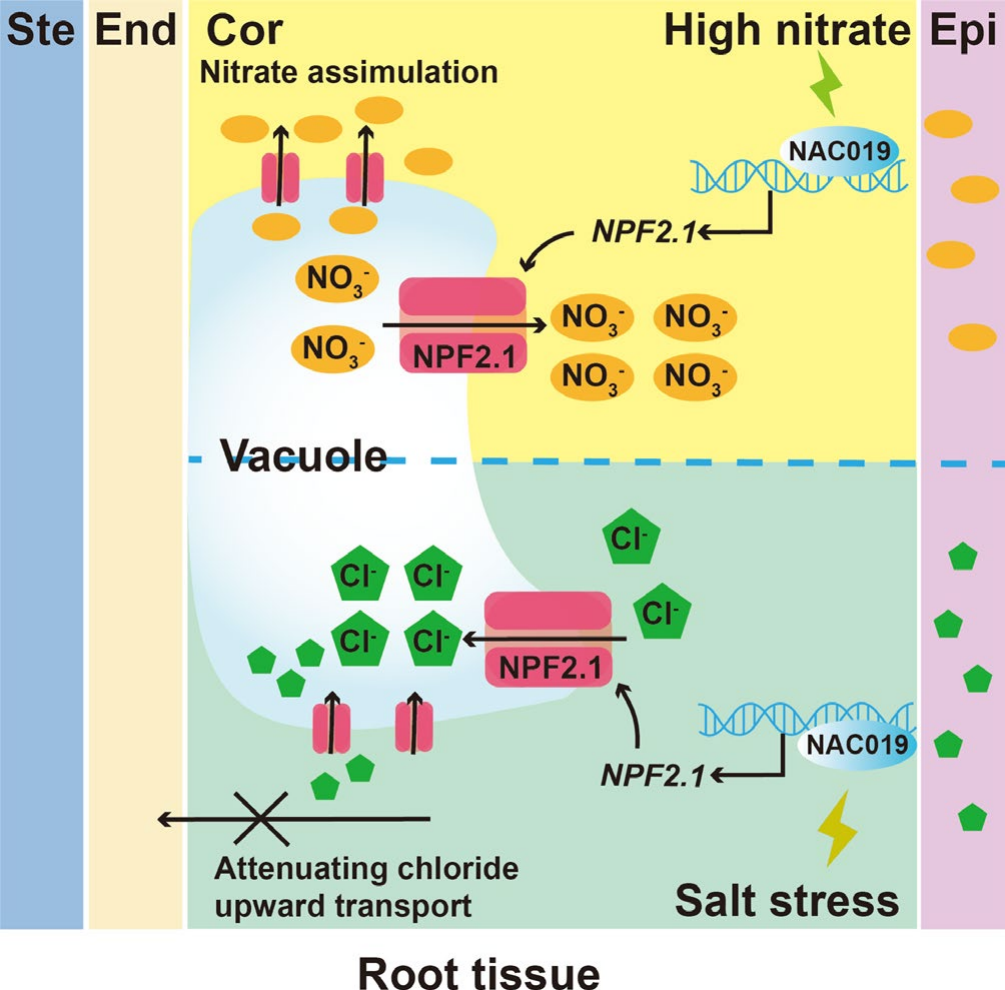
A dual‐function model of the CtrNAC019‐CtrNPF2.1 module in salt tolerance and nitrogen utilisation. Under high nitrate supplementation, CtrNAC019 triggers the expression of *CtrNPF2.1*, which facilitates the efflux of nitrate into the cytosol, thereby promoting nitrate assimilation. Under saline stress conditions, CtrNAC019 also activates the expression of *CtrNPF2.1*. Chloride influx into the root cortex is sequestered into the vacuole by CtrNPF2.1, leading to a reduction in chloride accumulation in the shoots. Cor, cortex; Epi, epidermis; End, endodermis; Ste., stele.

Based on these findings, we propose a working model to elucidate the mechanism by which the CtrNAC019‐CtrNPF2.1 module coordinates chloride sequestration and nitrogen utilisation in response to salt stress and high‐nitrate conditions (Figure 8). Under saline stress, CtrNAC019 responds to stress signals and activates the expression of *CtrNPF2.1*. CtrNPF2.1, which is anchored in the root cortex vacuole membrane, facilitates the sequestration of chloride ions into the vacuole, thereby reducing the upward transport of chloride ions. When plants sense an adequate supply of nitrogen, CtrNAC019 upregulates the transcriptional activity of *CtrNPF2.1*, promoting the release of nitrate from the vacuole to the cytosol and consequently enhancing nitrogen use efficiency. These findings provide novel insights into the understanding of nitrate and chloride transport mechanisms in plants under conditions of sufficient nitrogen and saline stress. The physiological characteristics of salt‐enhanced nitrate utilisation (SENU) and nitrogen‐mediated salt tolerance (NMST) can be elucidated from a molecular perspective through the CtrNAC019‐CtrNPF2.1 module. Taken together, this study identifies *CtrNPF2.1* as a promising candidate gene for molecular breeding, which can offer both enhanced saline resistance and nitrogen utilisation efficiency.

## 4 Materials and Methods

### 4.1 Plant Materials and Growth and Treatment Conditions

*Citrus trifoliata* seeds were collected from the Huazhong Agricultural University orchard (Wuhan, China). The seeds were germinated in darkness at 24°C and 65% relative humidity. Subsequently, the seedlings were grown in a hydroponic nutrient solution containing 2 mM KNO_3_, 1.5 mM Ca(NO_3_)_2_, 0.5 mM MgSO_4_, 0.14 mM Na_2_HPO_4_, 0.32 mM KH_2_PO_4_, 9.15 μM MnCl_2_, 1.6 μM ZnSO_4_, 0.32 μM CuSO_4_, 0.36 μM Na_2_MoO_4_, 37.3 μM Fe‐EDTA, and 10 μM H_3_BO_3_. All the root tissues were sampled for expression analysis. *A. thaliana* seeds were sown on 1/2 MS medium and subjected to vernalisation at 4°C for 24 h. Subsequently, the plates were transferred to a growth chamber for germination. After 14 days of seedlings growth, *Arabidopsis* plants were transplanted to soil. For salt stress treatments, specified concentrations of NaCl or KCl were added to the nutrient solution during trifoliate orange cultivation. The 100 mM NaCl solution was used for irrigation during the *Arabidopsis* salt treatment. Tobacco (*Nicotiana benthamiana*) seedlings were grown in a growth chamber set at 24°C with 65% relative humidity under a 16‐h photoperiod. Virus‐induced gene silencing was performed on trifoliate orange seedlings. The protocol references previous study (Dai et al. 2018).

### 4.2 *Agrobacterium* Mediated Plant Materials Transgenic Methods

For recombinant vector construction, the CDS sequences of *CtrNPF2.1* and *CtrNAC019* were cloned into the pK7WG2D vectors to generate overexpression constructs, while portions of the sequences were inserted into the pTRV2 vector (Hao et al. 2024) for silence of specific genes. *Agrobacterium* harbouring the correct plasmid was cultured in LB medium to an OD_600_ of 0.8, after which it was used for infection. The root‐hair transformation system utilized 3‐ to 4‐month‐old trifoliate orange seedlings. The roots of the uniformly developed seedlings were excised, and the cut portion was immersed in an infection solution prepared from the MES suspension. The samples were incubated for 15 min under vacuum and subsequently for 30 min under normal pressure. New roots were induced through cuttings, and positive roots exhibiting fluorescence were identified using a UV lamp, while non‐fluorescent roots were removed. The *Arabidopsis* transformation and homozygous line screen was performed as described (Wang and Tsay 2011). The virus‐induced gene silencing (VIGS) was carried out using trifoliate orange seedlings at the same growth stage as the experimental material. The seedlings were subjected to punctures on both root and shoot surfaces, subsequently immersed in a MES‐based infection solution. They were treated with vacuum infiltration for 5 min, followed by atmospheric pressure exposure for 10 min, dark incubation for 2 days, and finally transferred to soil for planting. Primers used for vector construction are listed in Table S1.

### 4.3 RNA Extraction, Reverse Transcription and RT‐qPCR Assay

The EASYspin Plant RNA Kit (Aidlab, Beijing, China) was employed for RNA extraction. Total RNA from each sample was reverse‐transcribed into cDNA using the TransScript IV One‐Step gDNA Removal and cDNA Synthesis SuperMix (TransGen, Beijing, China). RT‐qPCR analysis was performed on the Applied Biosystems QuantStudioTM 7 Flex Real‐Time PCR System (ABI, Waltham, MA, USA). All primers utilized in this study are detailed in Table S1.

### 4.4 RNA‐Seq Data Processing and Bioinformatics Analysis

The RNA sequencing was conducted on the Illumina platform by Novogene Technology Co. Ltd. (Nanjing, China). The raw data processing and gene annotation followed the methods described in a previously published study (Zhao et al. 2022). Gene Ontology (GO) and Kyoto Encyclopedia of Genes and Genomes (KEGG) enrichment analyses were conducted by TBtools (Chen et al. 2020). All genomic information pertaining to trifoliate was downloaded from the Citrus Pan‐genome to Breeding Database (CPBD, http://citrus.hzau.edu.cn/index.php).

### 4.5 In Situ Hybridisation Assay

*Citrus trifoliate* root samples were collected and washed with PBS, followed by immediate immersion in the fixative solution for in situ hybridisation for a minimum of 12 h. The roots of 2‐month‐old seedlings were fixed for in situ hybridisation assay. RNASweAMITM plant in situ hybridisation (BCIP/NBT) detection kit (Cat. GF011; Servicebio, Wuhan, China) was used to detect the specific localisation of CtrNPF2.1 in roots. Anti‐DIG‐AP antibody was then added and incubated at room temperature for 50 min, followed by 4‐time washes with PBS (5 min each) buffer. BCIP/NBT substrate solution was added until positive microscopic signals were observed, after washing with distilled water. Finally, nuclear fast red dye solution was used as a counterstain, followed by a final wash step.

### 4.6 Subcellular Localisation Assay

The coding sequence without stop codon of *CtrNPF2.1* or *CtrNAC019* was fused into the pRI101‐YFP vector and then transformed into *Agrobacterium* GV3101 for subcellular localisation assay. The transformation of tobacco leaves was performed using an infection solution prepared from the MES suspension. The solution was infiltrated into tobacco leaves, followed by a 12‐h incubation in the dark and a subsequent 48‐h period for gene expression. A confocal microscope (Leica TCS SP8, Mannheim, Germany) was utilized to observe the fluorescent signals of transgenic *N. benthamiana* leaves. To further confirm the localisation of the detected protein, the protoplasts were isolated to release the vacuoles according to the previously described method (Li et al. 2020). The YFP signal was excited at 488 nm, and the emitted signal was detected using a band‐pass filter (500–540 nm). Chlorophyll autofluorescence was excited at 552 nm, with the emission signal detected within the 590–650 nm range. The primers used in this study are provided in Table S1.

### 4.7 Synthesis of cRNA and Protein Expression in *X. laevis* Oocyte Cell

The *CtrNPF2.1* sequence was amplified and inserted into oocyte expression vectors (with or without the YFP construct) using a previously described advanced uracil excision‐based cloning technique (Li et al. 2020). The mMESSAGE mMACHINE kit (T7, Thermo Fisher, Waltham, MA, USA) was used for cRNA synthesis. A total of 50 nL of 400 ng/μL cRNA was injected into each healthy *X. laevis* oocyte for protein expression. After 2 days, the YFP signal in oocytes was detected by confocal microscopy (Leica TCS SP8, Mannheim, Germany) to ensure the protein was expressed on the plasma membrane.

### 4.8 Two‐Electrode Voltage Clamp (TEVC) Analysis of *X. laevis* Oocyte Cells

TEVC recordings were conducted on *X. laevis* oocytes injected with either ddH_2_O or *CtrNPF2.1* cRNA. The modified base solution (MBS) contained 6 mM Magnesium gluconate, 1.8 mM Calcium gluconate, and 10 mM MES, adjusted to pH 5.5 using Tris‐base propane, with osmolality adjusted to 210 mOsm through mannitol supplementation. For MBS with Cl^−^ or NO_3_^−^, the appropriate amount of hydrochloric acid or nitric acid was added before adjusting the pH value and osmotic pressure accordingly. Voltage step pulses ranging from +20 to −160 mV in −20 mV increments were applied to elicit currents. Whole‐cell current data were acquired and analysed using an AxoClamp 900A amplifier and pCLAMP software (version 11.2; Axon Instruments).

### 4.9 Absorption and Effluxion Assay of cl^−^ or ^15^NO_3_ ^−^ in *X. laevis* Oocyte Cells

To investigate the absorption activity of CtrNPF2.1, oocyte cells were injected with either water or cRNA and incubated in MBS containing 50 mM Cl^−^ or ^15^NO_3_^−^ for 2 h. Following this incubation period, the oocytes were washed and collected for elemental analysis. Each absorption sample contained 10 oocytes. In contrast, to assess efflux activity, oocytes were injected with 100 mM K^15^NO_3_ and KCl (injected 50 nL per cell) and incubated in MBS for 1 h before being washed and collected to determine the remaining elemental content. Each effluxion sample contained 4 oocytes. Three biological replicates were performed for each assay.

### 4.10 Stress Physiological and Root Scan Analysis

Chlorophyll fluorescence imaging was captured based on a chlorophyll fluorimeter (IMAGING‐PAM, Walz, Germany), and Fv/fm ratios were calculated by Imaging WinGegE software. To test the physiological parameters, 0.1 g plant sample was collected after treatment and then ground into powder with 2 mL PBS solution. The H_2_O_2_ content, O_2_^•‐^ content, and protein content were measured using commercially available kits (Jiancheng, Nanjing, China). Data for each indicator was obtained from at least three biological replicates. The evaluation of root growth parameters was conducted using an LD‐WinRHIZO plant root scanner (AgriPheno, Shanghai, China).

### 4.11 Element Content Assay

To determine the element content, plant samples were dried and then ground into powder after collection. Dried plant samples were accurately weighed at 0.1 g and then added to 50 mL of ddH_2_O. The mixture was stirred thoroughly and let it stand for 1 h. Subsequently, the solution was filtered using a funnel and the chloride content was determined using the Skalar flow injection analyser (Keaille Testing Technology Co. Ltd., Hubei, China). The nitrate or ^15^N‐labelled nitrate content was analysed by the Elementar Vario PYRO cube and Isoprime100 instruments (Elementar, Shanghai, China). Sodium and potassium contents were measured utilizing the Inductively Coupled Plasma‐Optical Emission Spectrometer (Agilent, American). Plant material samples were subjected to high‐temperature digestion with 3 mL H_2_SO_4_ and 1 mL H_2_O_2_, followed by dilution to 50 mL with ddH_2_O for element content assay.

### 4.12 Luciferase Complementation Imaging (LCI) Assay

The full length CDS of CtrNPF2.1 was cloned into the JW771‐LUC‐C vector and JW772‐LUC‐N vector. Next, these constructs were transfected into *A. tumefaciens* cells and combined in various pairings CtrNPF2.1‐JW771 and CtrNPF2.1‐JW772, and were designated as the experimental group. Each combination with a final optical density at OD600 of 0.2 in the transformation buffer (10 mmol/L 2‐[N‐morpholino]ethanesulfonic acid (MES) pH 5.6, 10 mmol/L MgCl_2_ and 150 μmol/L acetosyringone) was infiltrated into N. benthamiana leaves. Primers used in this study are listed in Table S1.

### 4.13 Bimolecular Fluorescence Complementation (BiFC) Assay

The full length CDS of CtrNPF2.1 without stop codon was cloned into the pYAC‐PYNE and pYAC‐PYCE vectors. All constructs were transiently expressed in tobacco leaves by *A. tumefaciens* ‐mediated transformation for over 48 h. Confocal microscopy of the released vacuole was performed with a Leica microscope (SP8, Leica, Mannheim, Germany). The YFP signal was excited at 514 nm and emitted a signal detected using a 520–540 nm band‐pass filter.

### 4.14 Yeast One Hybrid (Y1H) Assay

The 1504 bp promoter fragment of *CtrNPF2.1*, amplified from trifoliate orange genomic DNA, was inserted into the pAbAi vector and subsequently transformed into competent Y1H Gold yeast cells as bait. Potential transcription factor candidates were cloned into the pGADT7 vector to generate prey constructs, which were co‐transformed with the *CtrNPF2.1* promoter‐bait construct into Y1H Gold yeast cells. Yeast transformants were cultured on SD/‐Ura/−Leu medium either in the presence or absence of 150 ng/mL AbA for 3–5 days at 30°C. Positive (pGADT7‐p53 + p53‐AbAi) and negative (pGADT7‐AD + bait) controls were processed under identical conditions. The primers used in this study are provided in Table S1.

### 4.15 Dual Luciferase Reporter (LUC) Assay

The full‐length coding sequence of *CtrNAC019* was cloned into the pGreenII 62‐SK vector for generating the effector construct. The promoter region of *CtrNPF2.1* was inserted into the pGreenII 0800‐LUC vector for the reporter construct (Hellens et al. 2005). The effector constructs or the empty vector of 62‐SK, along with the reporter construct, were co‐transformed into tobacco plants. Dual luciferase activity was detected following the previously described method (Wu et al. 2016). When calculating the luciferase enzyme activity, the empty vector 62‐SK and the reporter line were normalized to 1. The primers used in this study are provided in Table S1.

### 4.16 Electrophoretic Mobility Shift Assay (EMSA)

EMSA was performed using a recombinant protein expressed in *E. coli* harbouring the pET32a vector containing the *CtrNAC019* coding sequence with the His tag. The DNA probes containing the wild‐type CGTG motif were Biotin‐labelled. While mutated core region probes changing CGTG to AAAA and competitor DNA fragments were non‐biotinylated. All these probes were synthesized by Shanghai Sangon Biotechnology (Shanghai, China) based on the Ctr*NPF2.1* promoter sequence. The probes were incubated with the fusion protein in a 10 μL reaction buffer for 30 min, with or without the addition of competitor probe. The reaction solution was subjected to electrophoresis on a 6.5% native polyacrylamide gel and subsequently transferred electrophoretically to nylon membranes (Biosharp, Hefei, China). Following UV cross‐linking, the migration of the biotin‐labelled probe on the membrane was detected using chemiluminescence (Thermo Fisher Scientific, Waltham, MA, USA). Probe sequence used in this study is provided in Table S1.

### 4.17 Chromatin Immunoprecipitation (ChIP)‐qPCR Assay

The ChIP‐qPCR assay was conducted according to the previously provided protocol (Ming et al. 2021) with some modifications. In this study, plant material was collected from the *CtrNAC019* overexpression line with a 3 × Flag tag in trifoliate roots. For the ChIP assay, 1 g of each transformed plant root and control root tissues was used to immunoprecipitate protein‐DNA complexes. Following reverse cross‐linking, DNA was extracted and subjected to RT‐qPCR analysis. The fold enrichment was calculated using the previously described method (Xiao et al. 2024). The primers used in this study are provided in Table S1.

## Author Contributions

Chunlong Li conceived and designed the research. Zeqi Zhao performed most of the experiments and analysed the data, Chengwei Yang, Xiangming Shang and Mengdi Li assisted with the experiments. Zeqi Zhao wrote the manuscript draft, Chunlong Li and Ji‐Hong Liu finalized writing and revision of the manuscript.

## Funding

This work was supported by the National Natural Science Foundation of China (32322073, U25A20687), Fundamental Research Funds for the Central Universities (2662025YLPY003), and the Huazhong Agricultural University (start‐up funding to Chunlong Li).

## Disclosure

Accession numbers: CtrNPF2.1: Pt3g001010; *CtrNAC019*: Pt1g011900.

## Conflicts of Interest

The authors declare no conflicts of interest.

## Supporting information


**Figure S1:** The element content of trifoliate orange seedlings after salt treatment.
**Figure S2:** The differential expression genes and enrichment analysis.
**Figure S3:** The expression assay of *CtrNPF2.1* under low nitrogen conditions.
**Figure S4:** The subcellular localisation of CtrNPF2.1 protein.
**Figure S5:** The GFP fluorescence images of overexpression transgenic seedlings and nitrate reductase (NR) activity assay in *CtrNPF2.1*‐OE and ‐TRV silenced plants.
**Figure S6:**. The physiological experiment of transgenic *Arabidopsis* with the overexpression of *CtrNPF2.1*.
**Figure S7:** The expression and localisation of CtrNPF2.1‐YFP fusion protein in oocyte.
**Figure S8:** The model illustration of CtrNPF2.1‐mediated transport directions for nitrate and chloride.
**Figure S9:** Electrophysiological characterisation of CtrNPF2.1 under varying substrate concentrations and pH conditions.
**Figure S10:**. The verification of CtrNPF2.1 self‐interaction.
**Figure S11:** The CtrNPF2.1 protein mutation sites in TM3 and TM6.
**Figure S12:** Selection of candidate transcription factors binding to the promoter of *CtrNPF2.1*.
**Figure S13:** The physiological experiment of *CtrNAC019*‐TRV plants.
**Figure S14:** The phenotype recovery assay of *CtrNAC019*‐TRV plants by overexpression of *CtrNPF2.1*.
**Figure S15:**. The physiological experiment of *CtrNAC019*‐OE plants.
**Figure S16:** The physiological experiment on salt‐enhanced nitrate utilisation (SENU) and nitrogen‐mediated salt tolerance (NMST) of *CtrNPF2.1*‐TRV plants.

**Table S1:** List of primers used in this study.

**File S1:** The differential expression gene list compared between KCl, or NaCl‐treated and control samples from transcriptome data.

## Data Availability

All data is available within the manuscript and its supporting materials.

## References

[pbi70686-bib-0001] Alfatih, A. , J. Zhang , Y. Song , et al. 2023. “Nitrate‐Responsive OsMADS27 Promotes Salt Tolerance in Rice.” Plant Communications 4: 100458. 36199247 10.1016/j.xplc.2022.100458 PMC10030316

[pbi70686-bib-0002] Baetz, U. , C. Eisenach , T. Tohge , E. Martinoia , and A. De Angeli . 2016. “Vacuolar Chloride Fluxes Impact Ion Content and Distribution During Early Salinity Stress.” Plant Physiology 172: 1167–1181. 27503602 10.1104/pp.16.00183 PMC5047071

[pbi70686-bib-0003] Brumós, J. , J. M. Colmenero‐Flores , A. Conesa , et al. 2009. “Membrane Transporters and Carbon Metabolism Implicated in Chloride Homeostasis Differentiate Salt Stress Responses in Tolerant and Sensitive Citrus Rootstocks.” Functional & Integrative Genomics 9: 293–309. 19190944 10.1007/s10142-008-0107-6

[pbi70686-bib-0004] Chen, C. J. , H. Chen , Y. Zhang , et al. 2020. “TBtools: An Integrative Toolkit Developed for Interactive Analyses of Big Biological Data.” Molecular Plant 13: 1194–1202. 32585190 10.1016/j.molp.2020.06.009

[pbi70686-bib-0005] Christianson, J. A. , I. W. Wilson , D. J. Llewellyn , and E. S. Dennis . 2009. “The Low‐Oxygen‐Induced NAC Domain Transcription Factor Affects Viability of *Arabidopsis* Seeds Following Low‐Oxygen Treatment.” Plant Physiology 149: 1724–1738. 19176720 10.1104/pp.108.131912 PMC2663757

[pbi70686-bib-0006] Corratgé‐Faillie, C. , and B. Lacombe . 2017. “Substrate (Un)specificity of *Arabidopsis* NRT1/PTR FAMILY (NPF) Proteins.” Journal of Experimental Botany 68: 3107–3113. 28186545 10.1093/jxb/erw499

[pbi70686-bib-0007] Crawford, N. M. , and A. D. M. Glass . 1998. “Molecular and Physiological Aspects of Nitrate Uptake in Plants.” Trends in Plant Science 3: 389–395.

[pbi70686-bib-0008] Dahro, B. , C. L. Li , and J.‐H. Liu . 2023. “Overlapping Responses to Multiple Abiotic Stresses in Citrus: From Mechanism Understanding to Genetic Improvement.” Horticulture Advances 1: 4.

[pbi70686-bib-0009] Dai, W. S. , M. Wang , X. Q. Gong , and J.‐H. Liu . 2018. “The Transcription Factor FcWRKY40 of Functions Positively in Salt Tolerance Through Modulation of Ion Homeostasis and Proline Biosynthesis by Directly Regulating and Homologs.” New Phytologist 219: 972–989. 29851105 10.1111/nph.15240

[pbi70686-bib-0010] Du, X. R. , M. X. Su , Y. Jiao , et al. 2022. “A Transcription Factor SlNAC10 Gene of *Suaeda liaotungensis* Regulates Proline Synthesis and Enhances Salt and Drought Tolerance.” International Journal of Molecular Sciences 23: 9625. 36077020 10.3390/ijms23179625 PMC9455740

[pbi70686-bib-0011] Geilfus, C. M. 2018. “Chloride: From Nutrient to Toxicant.” Plant and Cell Physiology 59: 877–886. 29660029 10.1093/pcp/pcy071

[pbi70686-bib-0013] Han, K. J. , Y. Zhao , Y. H. Sun , and Y. Li . 2023. “NACs, Generalist in Plant Life.” Plant Biotechnology Journal 21: 2433–2457. 37623750 10.1111/pbi.14161 PMC10651149

[pbi70686-bib-0014] Hao, X. L. , X. H. Long , H. Y. Zhao , et al. 2024. “CsABCG11.2 Mediates Theanine Uptake to Alleviate Cadmium Toxicity in Tea Plants ( *Camellia sinensis* ).” Horticulture Advances 2: 19.

[pbi70686-bib-0015] He, Y. N. , J. S. Peng , Y. Cai , et al. 2017. “Tonoplast‐Localized Nitrate Uptake Transporters Involved in Vacuolar Nitrate Efflux and Reallocation in *Arabidopsis* .” Scientific Reports 7: 6417. 28743909 10.1038/s41598-017-06744-5 PMC5526873

[pbi70686-bib-0064] Hellens, R. P. , A. C. Allan , E. N. Friel , et al. 2005. “Transient Expression Vectors for Functional Genomics, Quantification of Promoter Activity and RNA Silencing in Plants.” Plant Methods 1, no. 1. 10.1186/1746-4811-1-13. PMC1334188 16359558

[pbi70686-bib-0063] Hu, P. , K. Zhang , and C. Yang . 2018. “BpNAC012 Positively Regulates Abiotic Stress Responses and Secondary Wall Biosynthesis.” Plant Physiology 179, no. 2: 700–717. 10.1104/pp.18.01167. 30530740 PMC6426422

[pbi70686-bib-0017] Hualpa‐Ramirez, E. , E. C. Carrasco‐Lozano , J. Madrid‐Espinoza , et al. 2024. “Stress Salinity in Plants: New Strategies to Cope With in the Foreseeable Scenario.” Plant Physiology and Biochemistry 208: 108507. 38467083 10.1016/j.plaphy.2024.108507

[pbi70686-bib-0018] Iqbal, N. , S. Umar , and N. A. Khan . 2015. “Nitrogen Availability Regulates Proline and Ethylene Production and Alleviates Salinity Stress in Mustard ( *Brassica juncea* ).” Journal of Plant Physiology 178: 84–91. 25800225 10.1016/j.jplph.2015.02.006

[pbi70686-bib-0019] Katiyar‐Agarwal, S. , J. H. Zhu , K. M. Kim , et al. 2006. “The Plasma Membrane Na^+^/H^+^ Antiporter SOS1 Interacts With RCD1 and Functions in Oxidative Stress Tolerance in *Arabidopsis* .” Proceedings of the National Academy of Sciences of the United States of America 103: 18816–18821. 17023541 10.1073/pnas.0604711103 PMC1693745

[pbi70686-bib-0020] Krasensky, J. , and C. Jonak . 2012. “Drought, Salt, and Temperature Stress‐Induced Metabolic Rearrangements and Regulatory Networks.” Journal of Experimental Botany 63: 1593–1608. 22291134 10.1093/jxb/err460 PMC4359903

[pbi70686-bib-0065] Li, B. , C. Byrt , J. Qiu , et al. 2015. “Identification of a Stelar‐Localized Transport Protein That Facilitates Root‐to‐Shoot Transfer of Chloride in Arabidopsis.” Plant Physiology 170, no. 2: 1014–1029. 10.1104/pp.15.01163. 26662602 PMC4734554

[pbi70686-bib-0021] Li, B. , J. E. Qiu , M. Jayakannan , et al. 2017. “AtNPF2.5 Modulates Chloride (Cl^−^) Efflux From Roots of *Arabidopsis thaliana* .” Frontiers in Plant Science 7: 2013. 28111585 10.3389/fpls.2016.02013 PMC5216686

[pbi70686-bib-0022] Li, B. , M. Tester , and M. Gilliham . 2017. “Chloride on the Move.” Trends in Plant Science 22: 236–248. 28081935 10.1016/j.tplants.2016.12.004

[pbi70686-bib-0023] Li, C. L. , L. Dougherty , A. E. Coluccio , et al. 2020. “Apple ALMT9 Requires a Conserved C‐Terminal Domain for Malate Transport Underlying Fruit Acidity.” Plant Physiology 182: 992–1006. 31772076 10.1104/pp.19.01300 PMC6997694

[pbi70686-bib-0024] Li, H. , Y. Wang , L. Deng , and Z. Pan . 2025. “Microbial Biobank‐Based Strain Phenotyping Efficiently Identifies Plant Growth‐Promoting Bacteria From Citrus Rhizosphere.” Horticulture Advances 3: 3.

[pbi70686-bib-0025] Liu, X. X. , Y. X. Zhu , X. Z. Fang , et al. 2020. “Ammonium Aggravates Salt Stress in Plants by Entrapping Them in a Chloride Over‐Accumulation State in an NRT1.1‐Dependent Manner.” Science of the Total Environment 746: 141244. 32768787 10.1016/j.scitotenv.2020.141244

[pbi70686-bib-0026] Liu, Y. Q. , H. R. Wang , Z. M. Jiang , et al. 2022. “Genomic Basis of Geographical Adaptation to Soil Nitrogen in Rice.” Nature 590: 600–605. 10.1038/s41586-020-03091-w 33408412

[pbi70686-bib-0027] Lucas, M. , A. Diaz‐Espejo , D. Romero‐Jimenez , et al. 2024. “Chloride Reduces Plant Nitrate Requirement and Alleviates Low Nitrogen Stress Symptoms.” Plant Physiology and Biochemistry 212: 108717. 38761542 10.1016/j.plaphy.2024.108717

[pbi70686-bib-0028] Martínez‐Alcántara, B. , M. R. Martínez‐Cuenca , A. Quiñones , D. J. Iglesias , E. Primo‐Millo , and M. A. Forner‐Giner . 2015. “Comparative Expression of Candidate Genes Involved in Sodium Transport and Compartmentation in Citrus.” Environmental and Experimental Botany 111: 52–62.

[pbi70686-bib-0029] Mickelbart, M. V. , P. M. Hasegawa , and J. Bailey‐Serres . 2015. “Genetic Mechanisms of Abiotic Stress Tolerance That Translate to Crop Yield Stability.” Nature Reviews Genetics 16: 237–251. 10.1038/nrg3901 25752530

[pbi70686-bib-0030] Ming, R. H. , Y. Zhang , Y. Wang , M. Khan , B. Dahro , and J.‐H. Liu . 2021. “The JA‐Responsive MYC2‐Transcriptional Regulatory Module in Contributes to Cold Tolerance by Modulation of Glycine Betaine Biosynthesis.” New Phytologist 229: 2730–2750. 33131086 10.1111/nph.17063

[pbi70686-bib-0031] Moya, J. L. , A. Gómez‐Cadenas , E. Primo‐Millo , and M. Talon . 2003. “Chloride Absorption in Salt‐Sensitive *Carrizo citrange* and Salt‐Tolerant *Cleopatra mandarin* Citrus Rootstocks Is Linked to Water Use.” Journal of Experimental Botany 54: 825–833. 12554725 10.1093/jxb/erg064

[pbi70686-bib-0032] Parker, J. L. , and S. Newstead . 2014. “Molecular Basis of Nitrate Uptake by the Plant Nitrate Transporter NRT1.1.” Nature 507: 68–72. 24572366 10.1038/nature13116 PMC3982047

[pbi70686-bib-0033] Rajappa, S. , P. Krishnamurthy , H. Huang , et al. 2024. “The Translocation of a Chloride Channel From the Golgi to the Plasma Membrane Helps Plants Adapt to Salt Stress.” Nature Communications 15: 3978. 10.1038/s41467-024-48234-z PMC11087495 38729926

[pbi70686-bib-0034] Ramakrishna, P. , F. M. Gámez‐Arjona , E. Bellani , et al. 2025. “Elemental Cryo‐Imaging Reveals SOS1‐Dependent Vacuolar Sodium Accumulation.” Nature 637: 1228–1233. 39814877 10.1038/s41586-024-08403-y PMC11779634

[pbi70686-bib-0035] Ren, Z. J. , F. L. Bai , J. W. Xu , et al. 2021. “A Chloride Efflux Transporter, BIG RICE GRAIN 1, Is Involved in Mediating Grain Size and Salt Tolerance in Rice.” Journal of Integrative Plant Biology 63: 2150–2163. 34647689 10.1111/jipb.13178

[pbi70686-bib-0036] Rosales, M. A. , J. D. Franco‐Navarro , P. Peinado‐Torrubia , P. Díaz‐Rueda , R. Alvarez , and J. M. Colmenero‐Flores . 2020. “Chloride Improves Nitrate Utilization and NUE in Plants.” Frontiers in Plant Science 11: 442. 32528483 10.3389/fpls.2020.00442 PMC7264407

[pbi70686-bib-0037] Sakuraba, Y. , D. Kim , S. H. Han , et al. 2020. “Multilayered Regulation of Membrane‐Bound ONAC054 Is Essential for Abscisic Acid‐Induced Leaf Senescence in Rice.” Plant Cell 32: 630–649. 31911455 10.1105/tpc.19.00569 PMC7054035

[pbi70686-bib-0038] Shi, Y. J. , J. S. Luo , and Z. H. Zhang . 2023. “CHLORIDE CHANNEL‐b Mediates Vacuolar Nitrate Efflux to Improve Low Nitrogen Adaption in *Arabidopsis* .” Plant Physiology 194: 1234–1248. 10.1093/plphys/kiad438 37527482

[pbi70686-bib-0039] Song, C. B. , M. B. Wu , Y. Zhou , et al. 2022. “NAC‐Mediated Membrane Lipid Remodeling Negatively Regulates Fruit Cold Tolerance.” Horticulture Research 9: uhac039. 35531317 10.1093/hr/uhac039 PMC9071380

[pbi70686-bib-0040] Storey, R. , and R. R. Walker . 1999. “Citrus and Salinity.” Scientia Horticulturae 78: 39–81.

[pbi70686-bib-0041] Sun, J. , J. R. Bankston , J. Payandeh , T. R. Hinds , W. N. Zagotta , and N. Zheng . 2014. “Crystal Structure of the Plant Dual‐Affinity Nitrate Transporter NRT1.1.” Nature 507: 73–77. 24572362 10.1038/nature13074 PMC3968801

[pbi70686-bib-0042] Takasaki, H. , K. Maruyama , S. Kidokoro , et al. 2010. “The Abiotic Stress‐Responsive NAC‐Type Transcription Factor OsNAC5 Regulates Stress‐Inducible Genes and Stress Tolerance in Rice.” Molecular Genetics and Genomics 284: 173–183. 20632034 10.1007/s00438-010-0557-0

[pbi70686-bib-0043] Wang, Y. , Z. Yuan , J. Wang , et al. 2023. “The Nitrate Transporter NRT2.1 Directly Antagonizes PIN7‐Mediated Auxin Transport for Root Growth Adaptation.” Proceedings of the National Academy of Sciences of the United States of America 120: e2221313120. 37307446 10.1073/pnas.2221313120 PMC10288568

[pbi70686-bib-0044] Wang, Y. Y. , and Y. F. Tsay . 2011. “Nitrate Transporter NRT1.9 Is Important in Phloem Nitrate Transport.” Plant Cell 23: 1945–1957. 21571952 10.1105/tpc.111.083618 PMC3123939

[pbi70686-bib-0045] Wen, Z. , S. D. Tyerman , J. Dechorgnat , E. Ovchinnikova , K. S. Dhugga , and B. N. Kaiser . 2017. “Maize NPF6 Proteins Are Homologs of *Arabidopsis* CHL1 That Are Selective for Both Nitrate and Chloride.” Plant Cell 29: 2581–2596. 28887406 10.1105/tpc.16.00724 PMC5774558

[pbi70686-bib-0046] Wu, H. , B. Fu , P. P. Sun , C. Xiao , and J.‐H. Liu . 2016. “A NAC Transcription Factor Represses Putrescine Biosynthesis and Affects Drought Tolerance.” Plant Physiology 172: 1532–1547. 27663409 10.1104/pp.16.01096 PMC5100760

[pbi70686-bib-0047] Wu, H. , and Z. Li . 2019. “The Importance of cl^−^ Exclusion and Vacuolar cl^−^ Sequestration: Revisiting the Role of cl^−^ Transport in Plant Salt Tolerance.” Frontiers in Plant Science 10: 1418. 31781141 10.3389/fpls.2019.01418 PMC6857526

[pbi70686-bib-0048] Wu, Y. Z. , J. Y. Yuan , L. K. Shen , et al. 2025. “A Phosphorylation‐Regulated NPF Transporter Determines Salt Tolerance by Mediating Chloride Uptake in Soybean Plants.” EMBO Journal 44: 923–946. 39753952 10.1038/s44318-024-00357-1 PMC11790925

[pbi70686-bib-0049] Xi, Y. , Q. Q. Ling , Y. Zhou , X. Liu , and Y. X. Qian . 2022. “ZmNAC074, a Maize Stress‐Responsive NAC Transcription Factor, Confers Heat Stress Tolerance in Transgenic.” Frontiers in Plant Science 13: 986628. 36247610 10.3389/fpls.2022.986628 PMC9558894

[pbi70686-bib-0050] Xiao, Q. Y. , Y. Chen , C. W. Liu , et al. 2021. “MtNPF6.5 Mediates Chloride Uptake and Nitrate Preference in Roots.” EMBO Journal 40: e106847. 34523752 10.15252/embj.2020106847 PMC8561640

[pbi70686-bib-0051] Xiao, W. , Y. Zhang , Y. Wang , et al. 2024. “The Transcription Factor TGA2 Orchestrates Salicylic Acid Signal to Regulate Cold‐Induced Proline Accumulation in Citrus.” Plant Cell 37: koae290. 39656997 10.1093/plcell/koae290 PMC11663595

[pbi70686-bib-0052] Xie, C. T. , C. L. Li , F. X. Wang , et al. 2023. “NAC1 Regulates Root Ground Tissue Maturation by Coordinating With the SCR/SHR‐CYCD6;1 Module in *Arabidopsis* .” Molecular Plant 16: 709–725. 36809880 10.1016/j.molp.2023.02.006

[pbi70686-bib-0053] Xu, Q. , L. L. Chen , X. A. Ruan , et al. 2013. “The Draft Genome of Sweet Orange ( *Citrus sinensis* ).” Nature Genetics 45: 59–66. 23179022 10.1038/ng.2472

[pbi70686-bib-0054] Yang, Y. , and Y. Guo . 2018. “Elucidating the Molecular Mechanisms Mediating Plant Salt‐Stress Responses.” New Phytologist 217: 523–539. 29205383 10.1111/nph.14920

[pbi70686-bib-0055] Zelm, E. v. , Y. X. Zhang , and C. Testerink . 2020. “Salt Tolerance Mechanisms of Plants.” Annual Review of Plant Biology 71: 403–433. 10.1146/annurev-arplant-050718-100005 32167791

[pbi70686-bib-0056] Zhang, X. G. , and B. Huang . 2019. “Prediction of Soil Salinity With Soil‐Reflected Spectra: A Comparison of Two Regression Methods.” Scientific Reports 9: 5067. 30911024 10.1038/s41598-019-41470-0 PMC6434016

[pbi70686-bib-0057] Zhao, C. , O. Zayed , Z. Yu , et al. 2018. “Leucine‐Rich Repeat Extensin Proteins Regulate Plant Salt Tolerance in *Arabidopsis* .” Proceedings of the National Academy of Sciences of the United States of America 115: 13123–13128. 30514814 10.1073/pnas.1816991115 PMC6305001

[pbi70686-bib-0058] Zhao, Z. Q. , M. D. Li , W. W. Xu , J.‐H. Liu , and C. L. Li . 2022. “Genome‐Wide Identification of NRT Gene Family and Expression Analysis of Nitrate Transporters in Response to Salt Stress in *Poncirus trifoliata* .” Genes 13: 1115. 35885900 10.3390/genes13071115 PMC9323722

[pbi70686-bib-0059] Zhou, H. P. , H. F. Shi , Y. Q. Yang , et al. 2023. “Insights Into Plant Salt Stress Signaling and Tolerance.” Journal of Genetics and Genomics 51: 16–34. 37647984 10.1016/j.jgg.2023.08.007

[pbi70686-bib-0060] Zhu, J. K. 2002. “Salt and Drought Stress Signal Transduction in Plants.” Annual Review of Plant Biology 53: 247–273. 10.1146/annurev.arplant.53.091401.143329 PMC3128348 12221975

[pbi70686-bib-0061] Zhu, J. K. 2016. “Abiotic Stress Signaling and Responses in Plants.” Cell 167: 313–324. 27716505 10.1016/j.cell.2016.08.029 PMC5104190

