# Supplementary material for: The Dual‐Function of CtrNAC019‐CtrNPF2.1 Module in Salt Tolerance and Nitrogen Use Efficiency Via Enhancing Vacuolar Chloride Sequestration and Nitrate Efflux in Citrus trifoliata
Source: Plant Biotechnol J. 2026 May 18;24(9):5059–76. doi: 10.1111/pbi.70686 (PMC13398927; doi:10.1111/pbi.70686)
Supplement: Supplementary file 1 [file PBI-24-5059-s002.pdf]

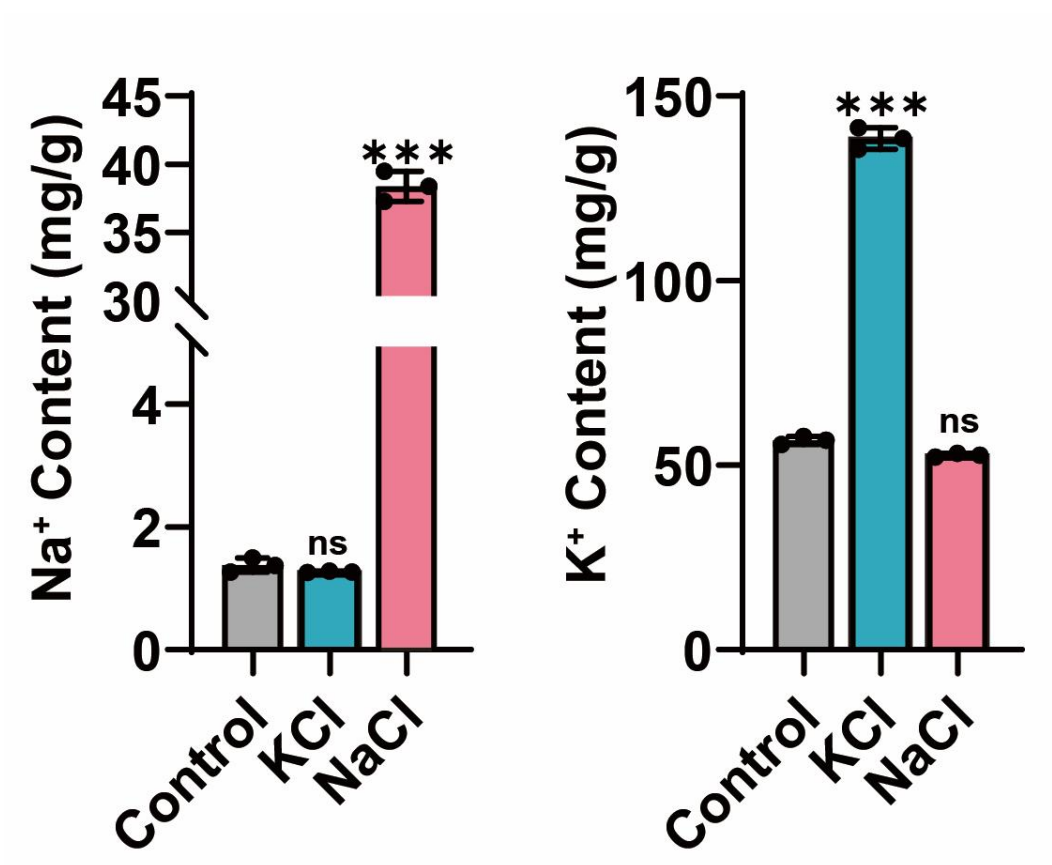

Supplementary Figure 1. The element content of trifoliate orange seedlings after salt treatment. (A-B) Sodium **(A)** and potassium **(B)** content was detected. The sample was collected from whole plant of Figure 1A. Values represent the mean ± SE with three biological replicates. The asterisks indicate significant differences as assessed by independent samples t-test, ns, $P > 0.05$, $***P < 0.001$.

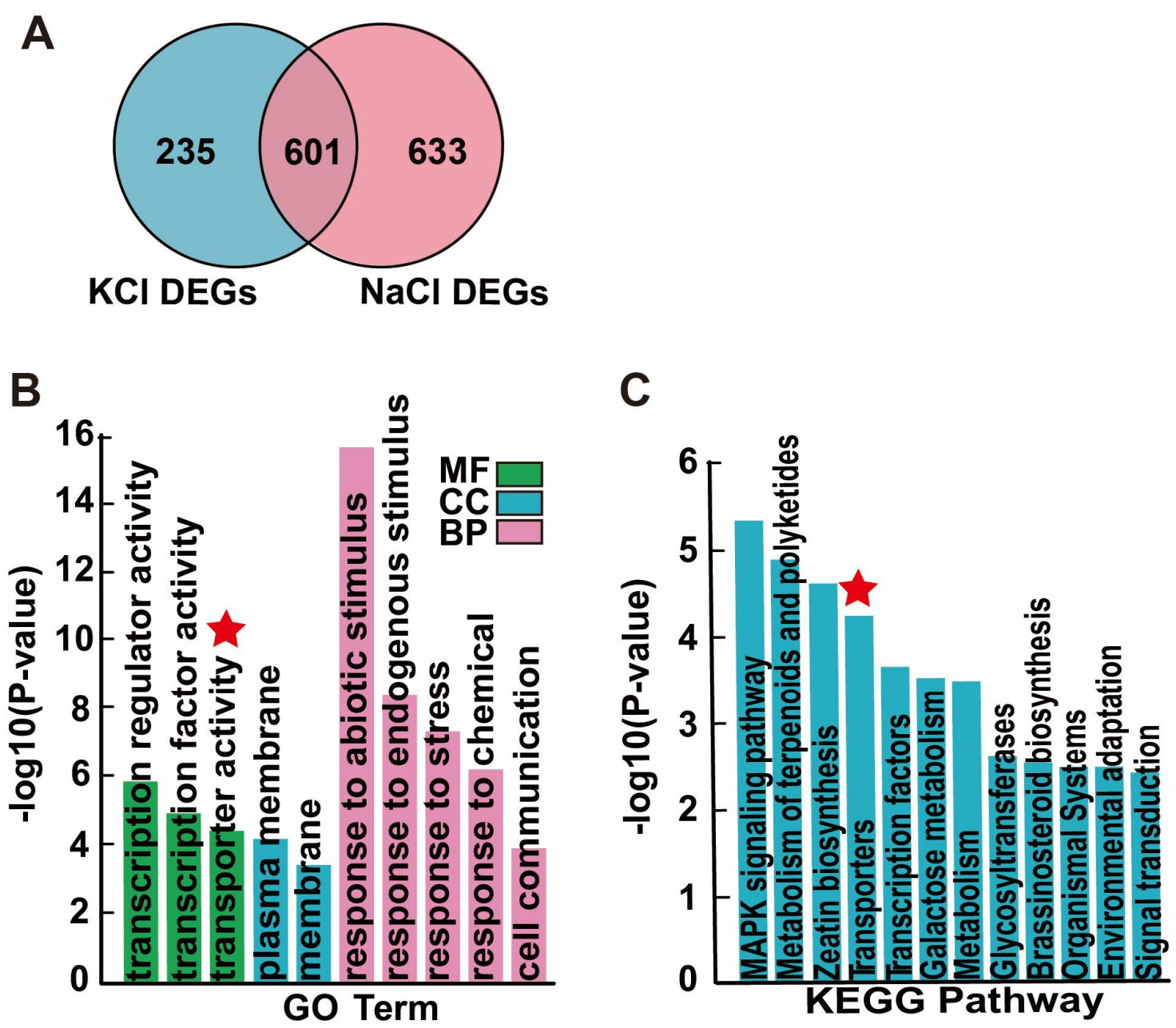

Supplementary Figure 2. The differential expression genes and enrichment analysis. (A) Venn diagram showing differential expression genes (DEGs) in KCl and NaCl-treated samples compared to control. **(B-C)** Enrichment analysis. GO **(B)** and KEGG **(C)** enrichment plots involving 601 genes represent the central intersection region of Venn diagram. MF, BP, and CC stand for molecular function, biology process, and cell component, respectively.

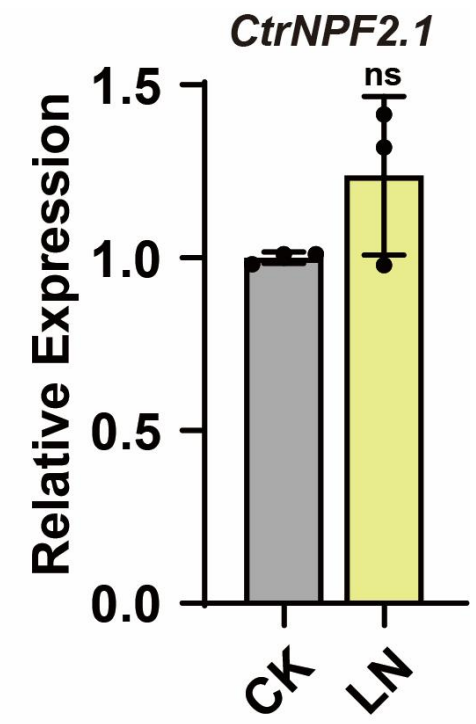

Supplementary Figure 3. The expression assay of *CtrNPF2.1* under low nitrogen conditions. The expression of *CtrNPF2.1* under normal (CK) and 0.1-fold (0.5 mM NO_3^-) low nitrate (LN) condition. Values represent the mean ± SE with three biological replicates. The asterisks indicate significant differences as assessed by independent samples t-test, ns, $P > 0.05$.

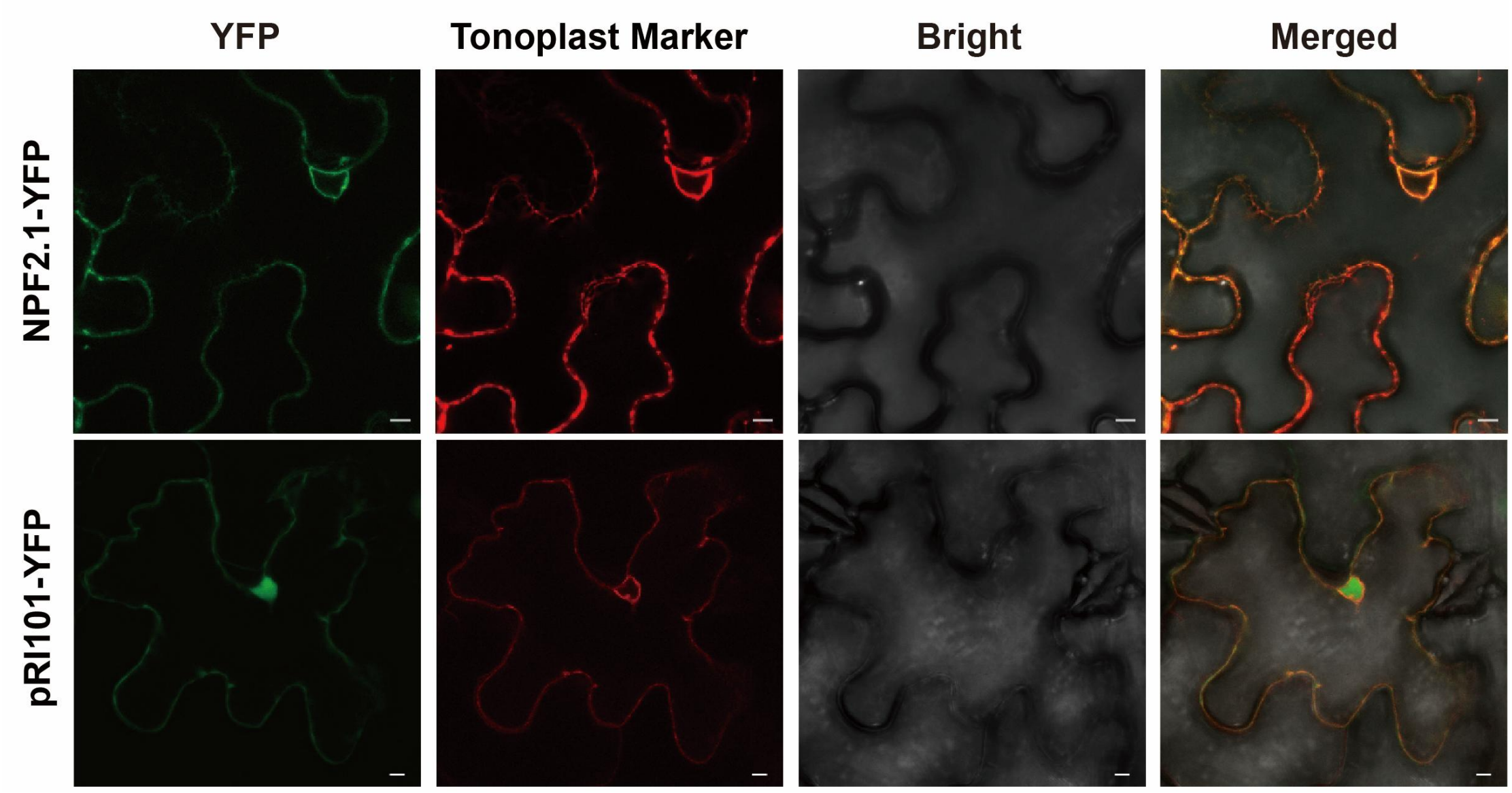

Supplementary Figure 4. The subcellular localization of CtrNPF2.1 protein. Images of confocal microscopy were epidermal cells expressing the empty vector (EV) control and CtrNPF2.1 tagged with YFP fluorescent proteins in *N. benthamiana*, respectively. Scale bars = 5 μm.

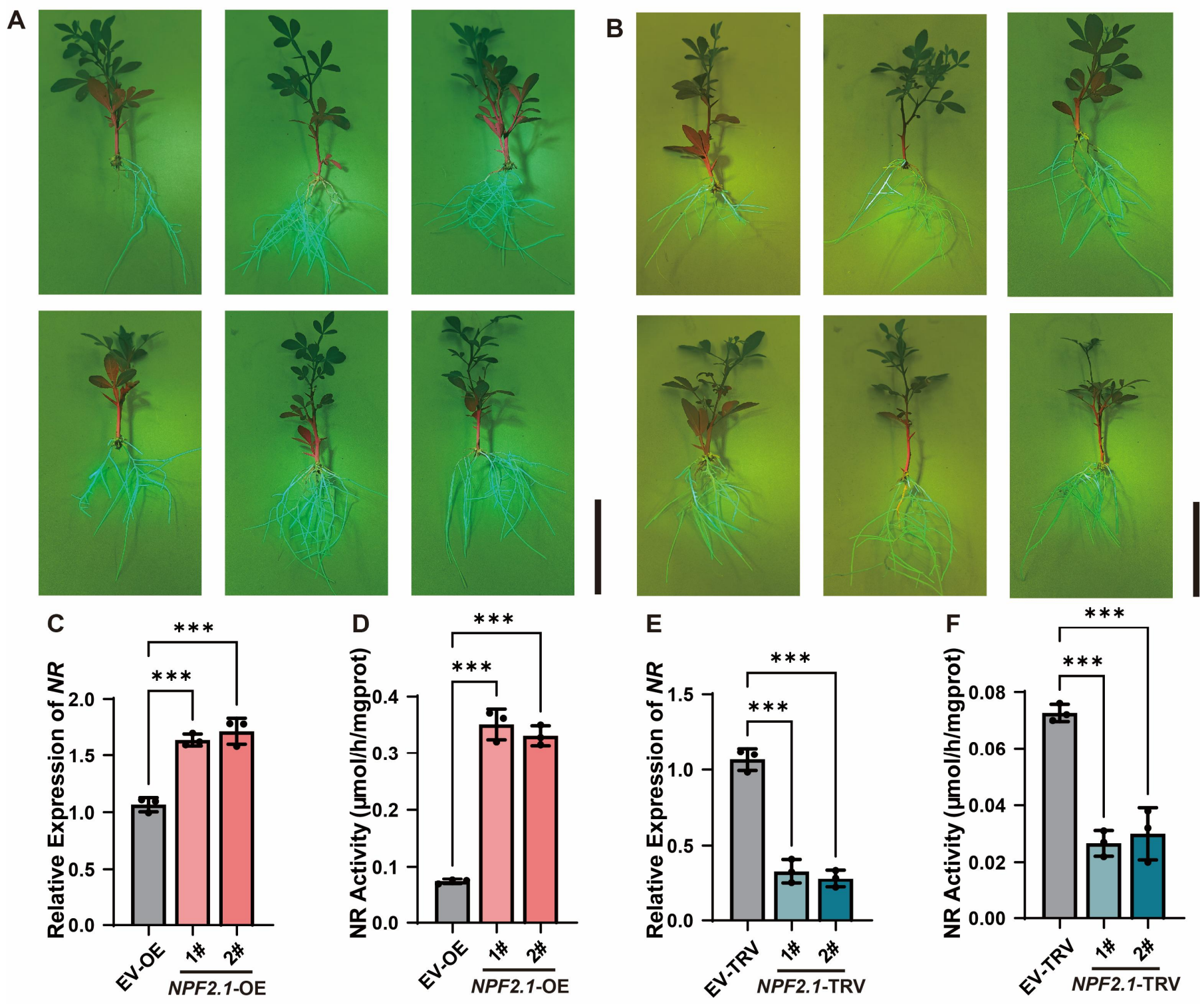

Supplementary Figure 5. The GFP fluorescence images of overexpression transgenic seedlings and nitrate reductase (NR) activity assay in *CtrNPF2.1*-OE and -TRV silenced plants. (A) Left: EV-OE; Middle: *CtrNPF2.1*-OE-1; Right: *CtrNPF2.1*-OE-2. **(B)** Left: EV-OE; Middle: *CtrNAC019*-OE-1; Right: *CtrNAC019*-OE-2. Scale bars = 10 cm. **(C-F)** The *NR* expression analysis and NR enzyme activity test in *CtrNPF2.1*-OE **(C, D)** and -TRV silenced **(E, F)** plants. Values represent the mean ± SE with three biological replicates. The asterisks indicate significant differences as assessed by independent samples t-test, ***$P < 0.001$.

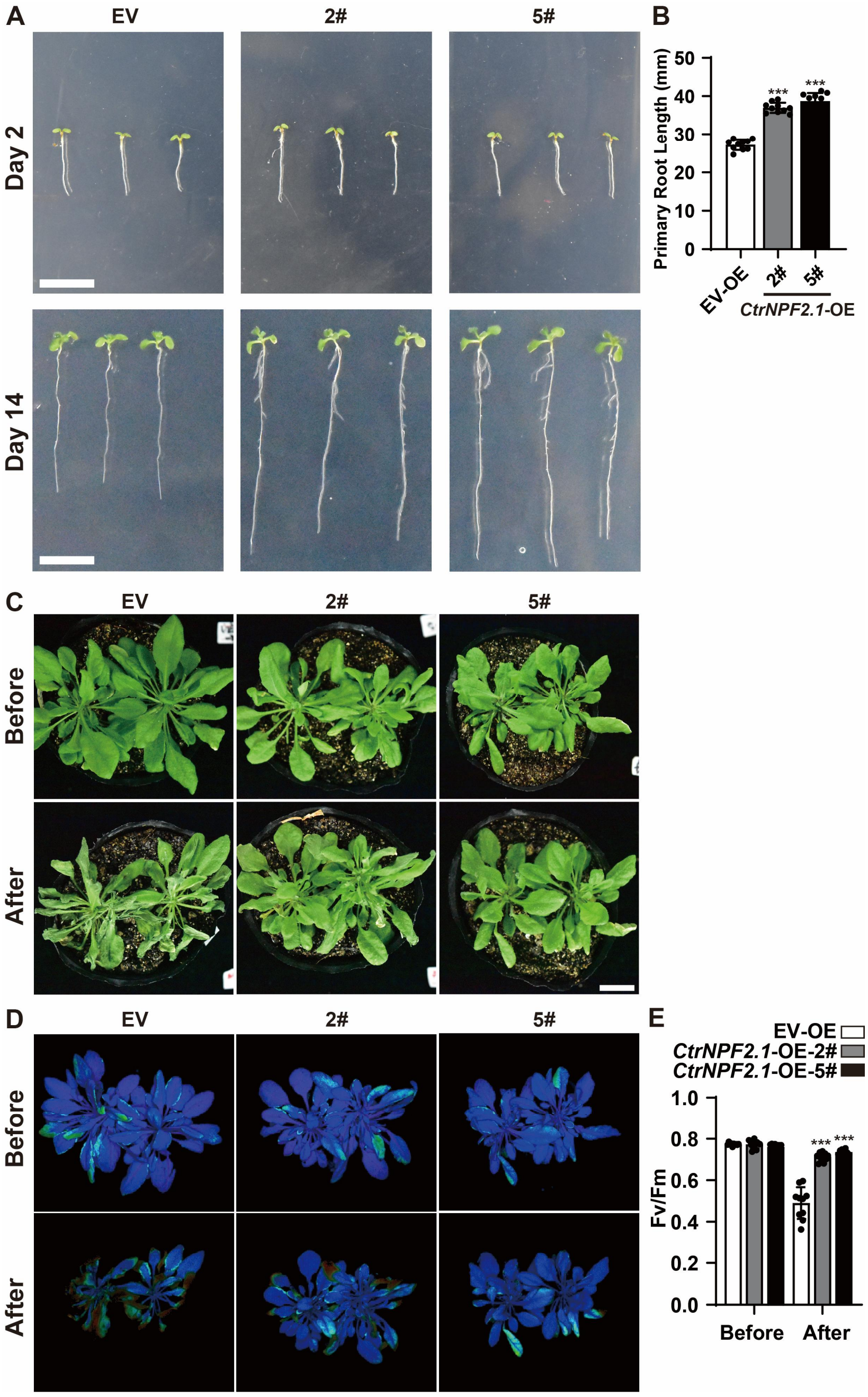

Supplementary Figure 6. The physiological experiment of transgenic *Arabidopsis* with the overexpression of *CtrNPF2.1*. (A) The root growth phenotype of *CtrNPF2.1*-OE transgenic *Arabidopsis* seedlings under high nitrogen conditions. Scale bars = 10 mm. **(B)** The primary root length detected at Day 14 as shown in panel A. **(C)** Salt stress was performed using 1-month old *CtrNPF2.1*-OE transgenic *Arabidopsis* seedlings. Scale bars = 20 mm. **(D-E)** Chlorophyll fluorescence imaging **(D)** and Fv/Fm **(E)** ratios of *CtrNPF2.1*-OE *Arabidopsis* seedlings before and after 4 days treatment with salt stress. Values represent the mean ± SE with three biological replicates. The asterisks indicate significant differences as assessed by independent samples t-test, $***P < 0.001$.

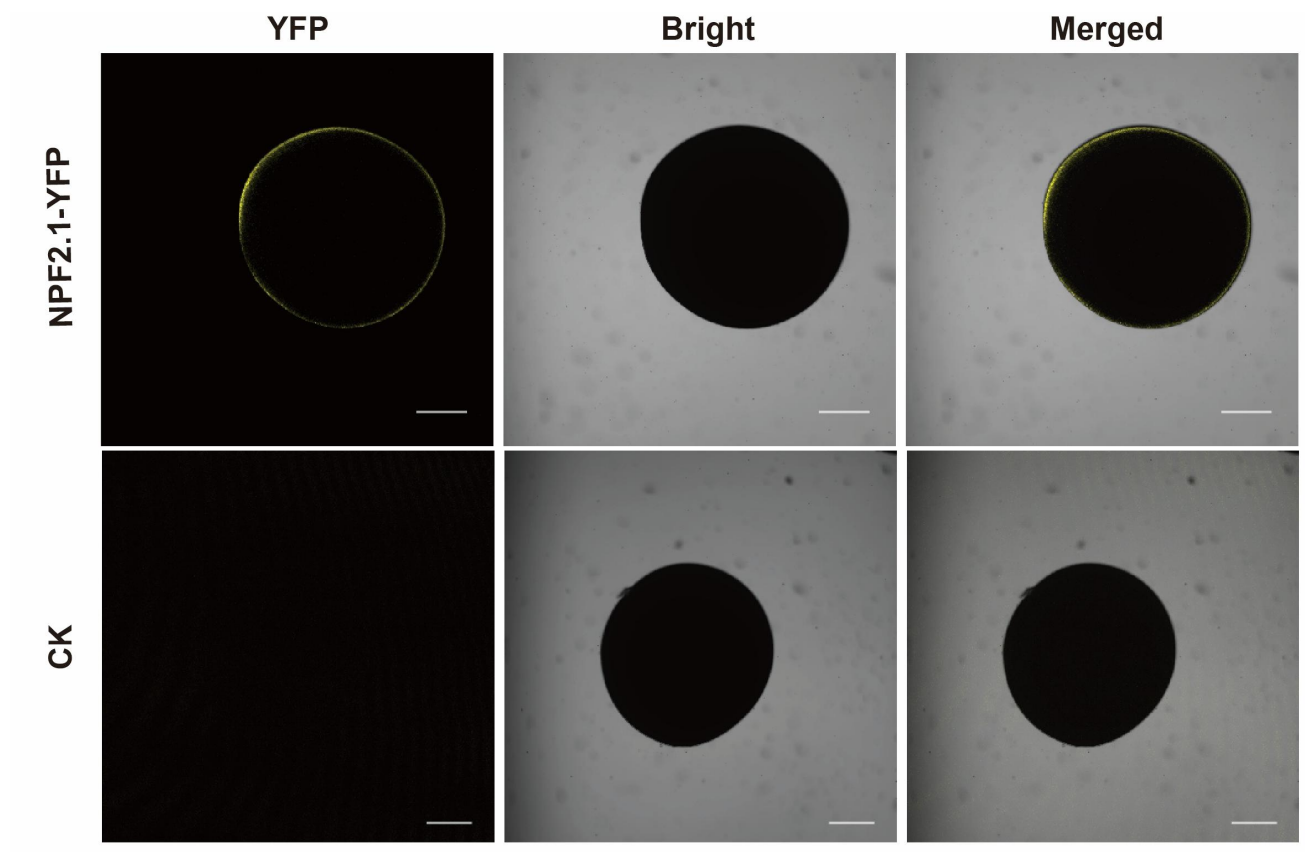

Supplementary Figure 7. The expression and localization of CtrNPF2.1-YFP fusion protein in oocyte. Images of confocal microscopy were *X. laevis* oocyte cell expressing CtrNPF2.1 tagged with YFP fluorescent proteins and the cell injected with water as the negative control. Scale bars = 200 μm.

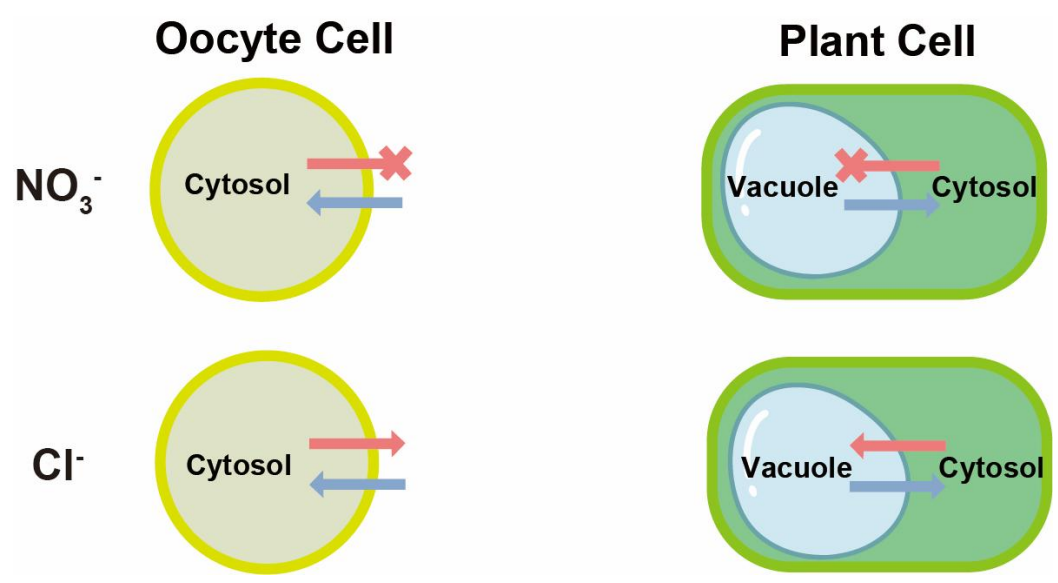

Supplementary Figure 8. The model illustration of CtrNPF2.1-mediated transport directions for nitrate and chloride. The models illustrate that CtrNPF2.1 mediates nitrate influx across the plasma membrane of oocytes and nitrate efflux across the tonoplast of plant cells (above). CtrNPF2.1 can dually transport chloride across both the oocyte plasma membrane and the plant tonoplast (below). The red line represents oocyte efflux and vacuolar influx, whereas the blue line represents oocyte influx and vacuolar efflux.

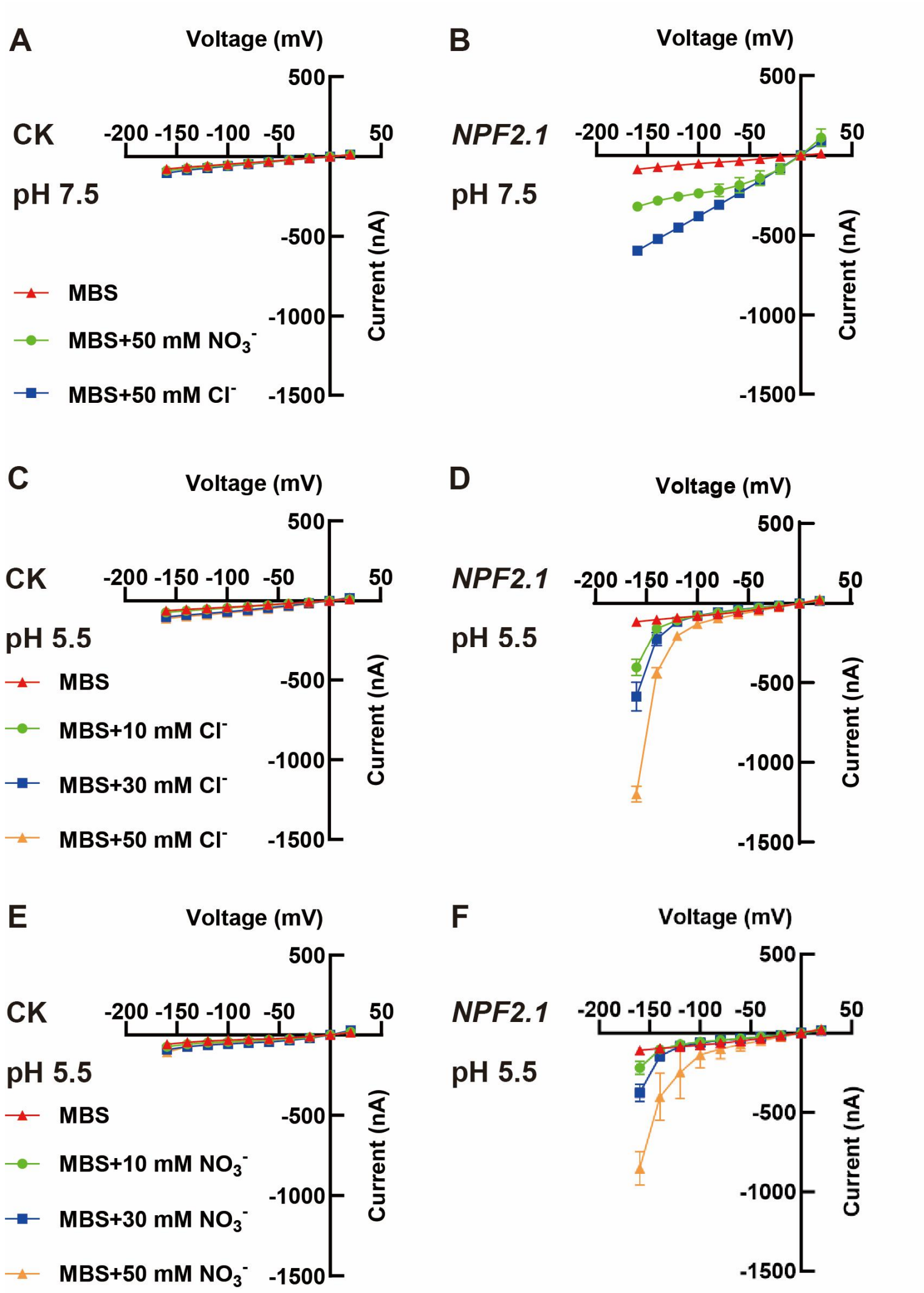

Supplementary Figure 9. Electrophysiological characterization of CtrNPF2.1 under varying substrate concentrations and pH conditions. (A-B) Current-voltage (I-V) relationship of *X. laevis* oocyte cells injected with water **(A)** and *CtrNPF2.1* cRNA **(B)**, n=4. The Cl^- and NO_3^- concentrations in MBS were 50 mM. The MBS solutions had a pH of 7.5 and an osmolality of 210 mOsm. **(C-D)** Cl^--elicited currents mediated by CtrNPF2.1 were dependent on external Cl^- concentrations, n=4. Oocytes injected with water **(C)** or *CtrNPF2.1* cRNA **(D)** were exposed to MBS containing 10-50 mM Cl^- at pH 5.5. **(E-F)** NO_3^--elicited currents mediated by CtrNPF2.1 were dependent on external NO_3^- concentrations, n=4. Oocytes injected with water **(E)** or *CtrNPF2.1* cRNA **(F)** were exposed to MBS containing 10-50 mM NO_3^- at pH 5.5. The MBS solutions had an osmolality of 210 mOsm. The experiment was repeated three times, with similar results.

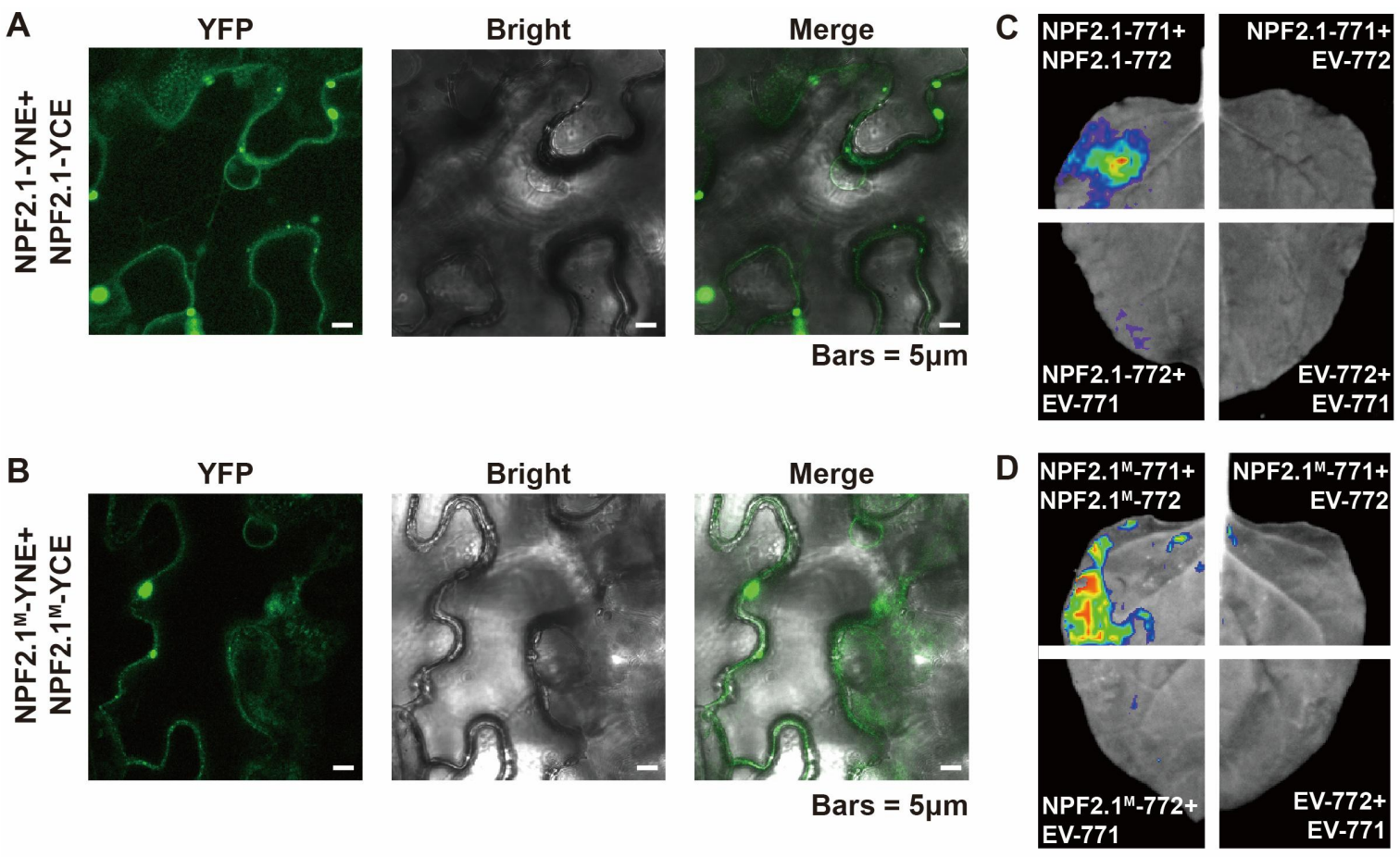

Supplementary Figure 10. The verification of CtrNPF2.1 self-interaction. (A-B) BiFC assays indicated that CtrNPF2.1 **(A)** or CtrNPF2.1 site mutant (CtrNPF2.1^{M}) **(B)** could interact itself. Co-expression of CtrNPF2.1-YNE (N-terminal GFP fusion) and CtrNPF2.1-YCE (C-terminal GFP fusion) or CtrNPF2.1^{M}-YNE and CtrNPF2.1^{M}-YCE in tobacco leaves was observed under confocal microscopy. **(C-D)** LCI assays demonstrated the *in vivo* self-interaction of CtrNPF2.1 or CtrNPF2.1^{M}. The infiltrated leaves contained the specified combinations of constructs as designated. Co-expression of CtrNPF2.1-771 (N-terminal LUC fusion) + CtrNPF2.1-772 (C-terminal LUC fusion) **(C)**, and CtrNPF2.1^{M}-771 + CtrNPF2.1^{M}-772 **(D)** served as the experimental group. CtrNPF2.1-771/JW772, JW771/CtrNPF2.1-772, and JW771/JW772 were used as negative controls.

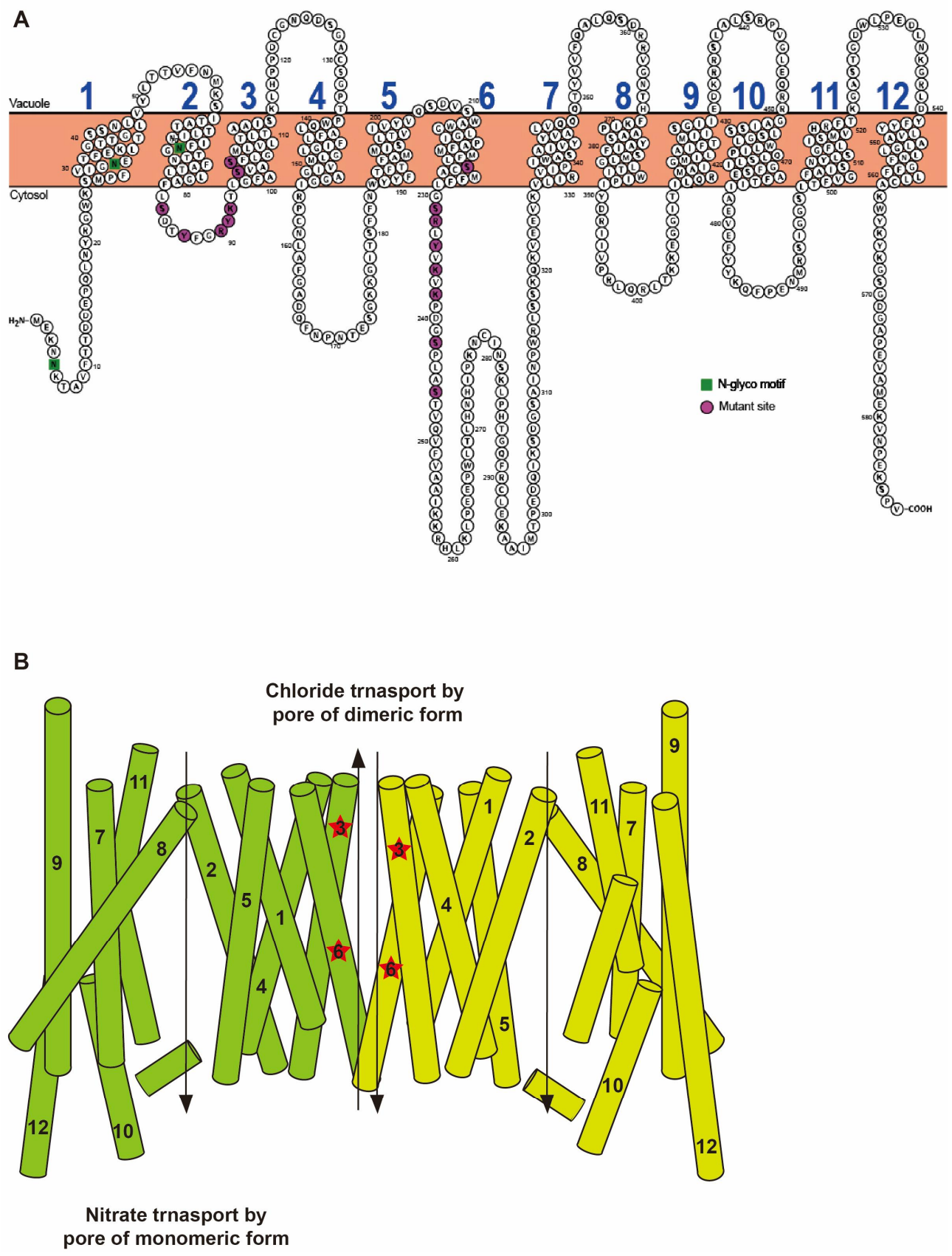

Supplementary Figure 11. The CtrNPF2.1 protein mutation sites in TM3 and TM6. **(A)** The transmembrane domain architecture of the CtrNPF2.1 protein, along with putative functional residues, may facilitate chloride transport, as visualized using PROTTER (http://wlab.ethz.ch/protter/start/). The purple sites were mutated to Ala. **(B)** Cartoon schematic diagram of CtrNPF2.1 dimer refereeing paper of Sun, J., et al. (http://dx.doi.org/10.1038/nature13074) with suspected chloride channel formed by TM3 and TM6.

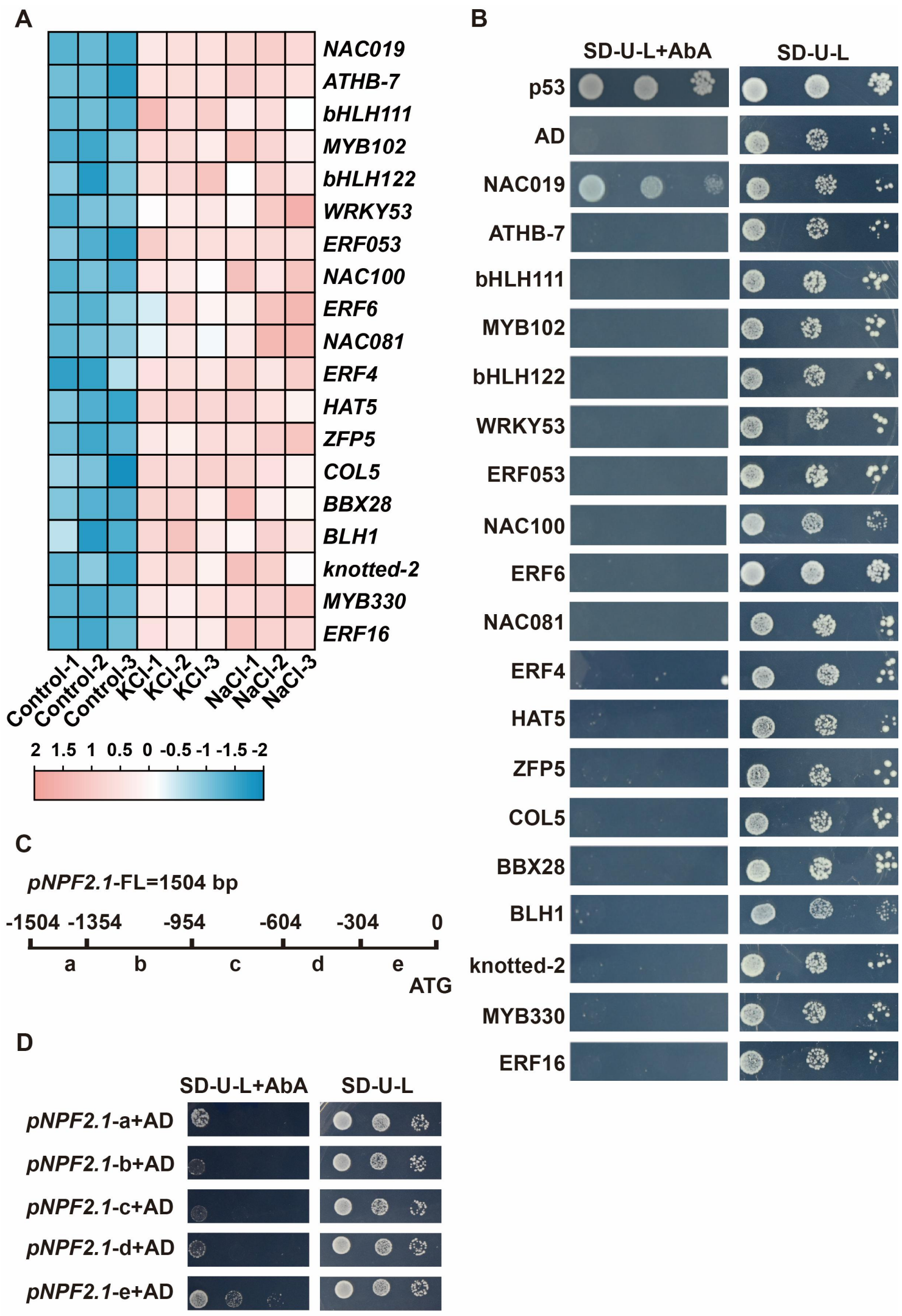

Supplementary Figure 12. Selection of candidate transcription factors binding to the promoter of *CtrNPF2.1*. (A) The 19 up-regulated transcription factors (TFs) in response to NaCl and KCl were selected for Y1H assay. **(B)** The yeast growth assay based on yeast one-hybrid (Y1H) system. Yeast cells co-transformed with different combinations of prey and baits on SD/-Ura/-Leu medium added with 0 or 150 ng/mL AbA. TFs-pGADT7 was used as a prey, while the *CtrNPF2.1* promoter fused to pAbAi plasmid was used as bait. The pGADT7 empty vector was used as negative control, and p53-AbAi + pGAD-p53 was used as positive control. The yeast with $OD_{600} = 0.2$ was diluted into 10^{-1}, 10^{-2}, 10^{-3}. **(C)** The schematic diagram of *CtrNPF2.1* promoter fragments. **(D)** The negative control of segmentation Y1H assay corresponding to Figure 5.

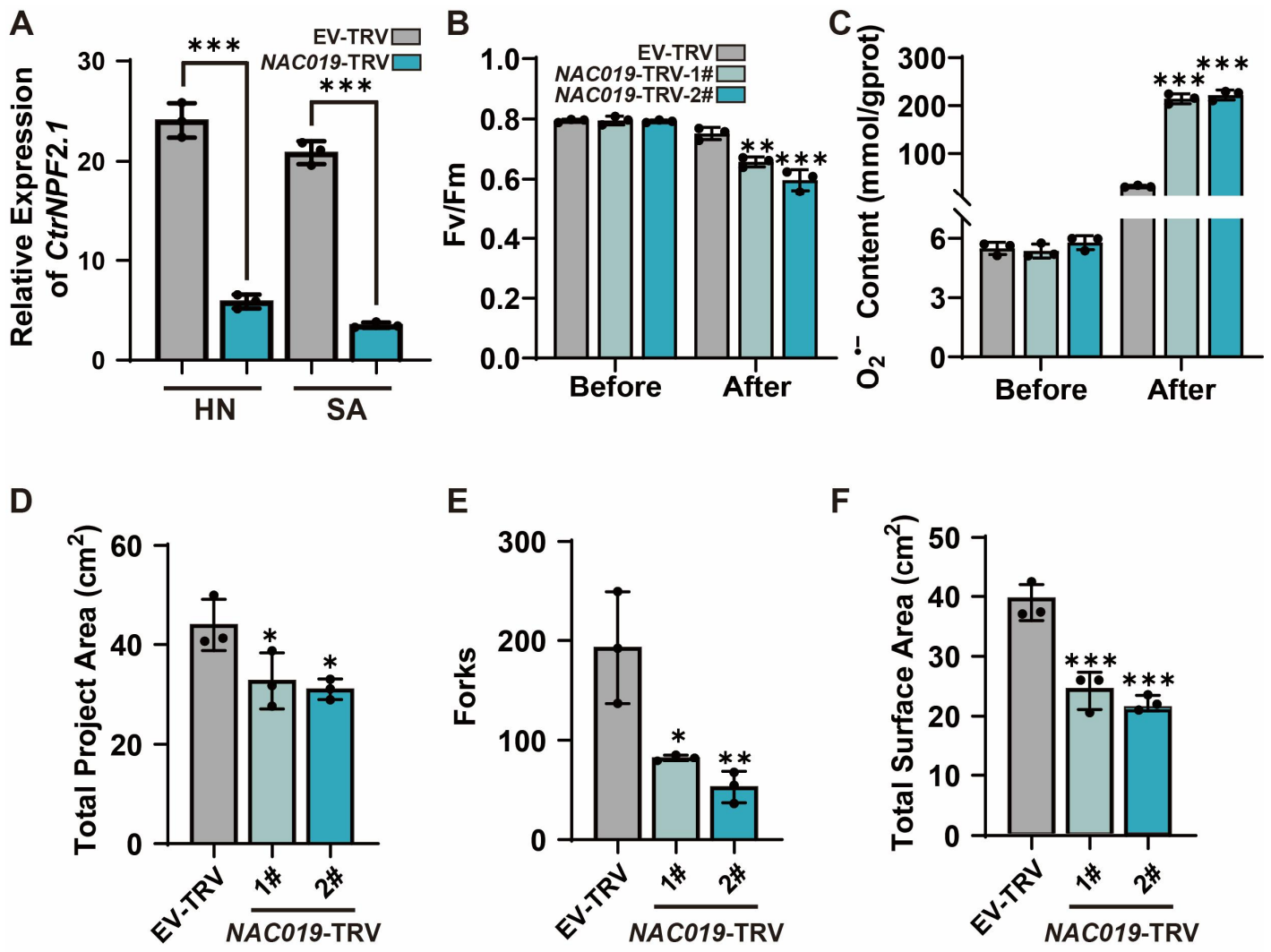

Supplementary Figure 13. The physiological experiment of *CtrNAC019*-TRV plants. **(A)** The expression level of *CtrNPF2.1* in *CtrNAC019*-TRV and EV-TRV materials under high nitrate (HN, 50 mM nitrate) and salt stress (SA, 200 mM NaCl) conditions. **(B)** The fluorescence of chlorophyll before and after treatment were detected response to Figure 6B. **(C)** The $O_2^{\bullet-}$ content in seedlings was measured before and after salt stress to quantify the degree of stress-induced damage. **(D-F)** The root parameter indicated the growth tendency conducting by root scanner, containing total project area **(D)**, forks **(E)**, and total surface area **(F)**. Values represent the mean ± SE with three biological replicates. The asterisks indicate significant differences as assessed by independent samples t-test, * 0.05< P < 0.01, ** 0.01< P < 0.001, ***P < 0.001.

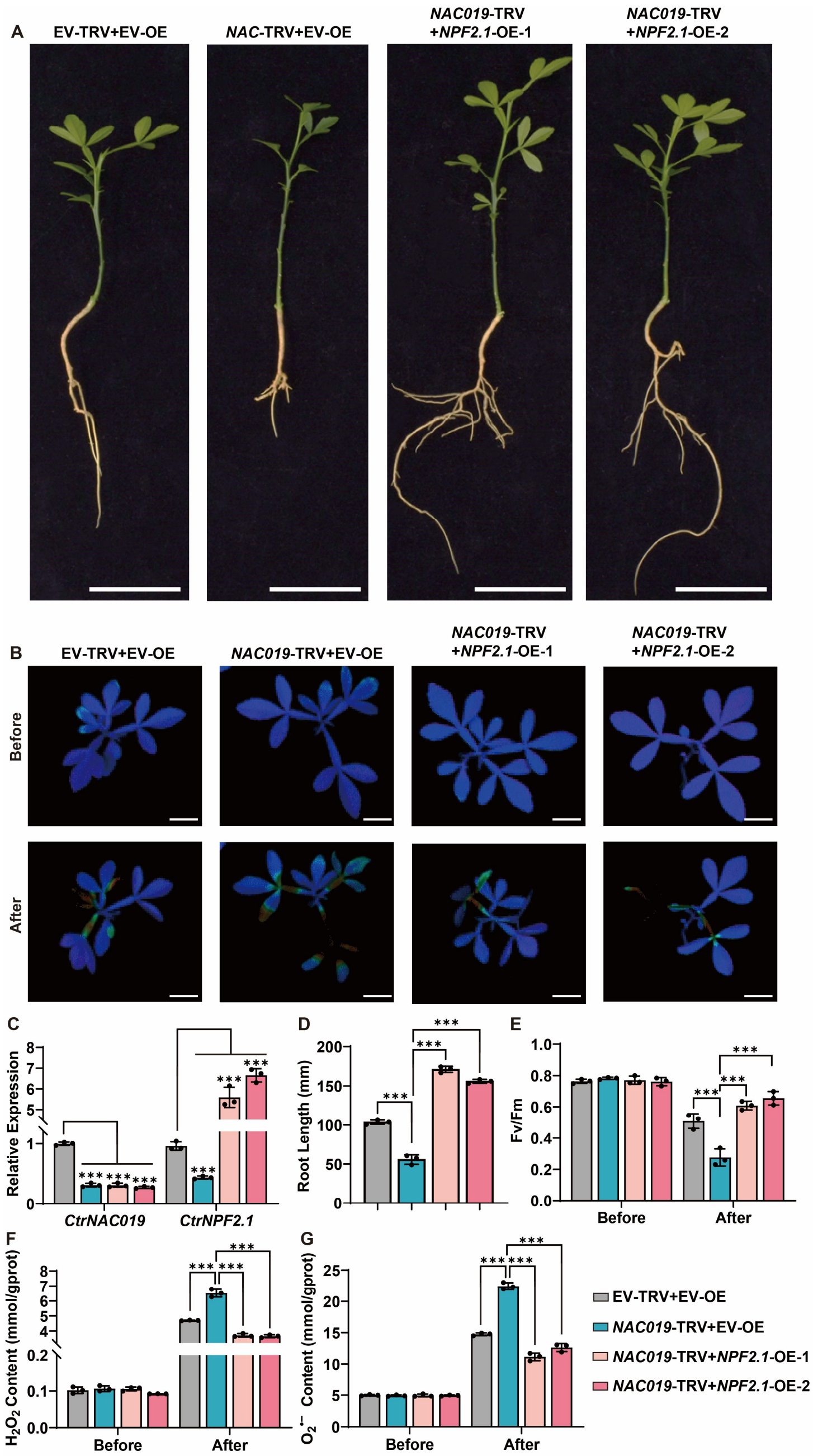

Supplementary Figure 14. The phenotype recovery assay of *CtrNAC019*-TRV plants by overexpression of *CtrNPF2.1*. (A) Root photograph in EV-TRV+EV-OE, *NAC019*-TRV+EV-OE, *NAC019*-TRV+*NPF2.1*-OE-1 and *NAC019*-TRV+*NPF2.1*-OE-2 plants. Scale bars = 5 cm. **(B)** Chlorophyll fluorescence imaging before and after 200 mM salt treatment of indicated plants. Scale bars = 1 cm. **(C)** The expression level of *NAC019* and *NPF2.1* were test in EV-TRV+EV-OE, *NAC019*-TRV+EV-OE, *NAC019*-TRV+*NPF2.1*-OE-1 and *NAC019*-TRV+*NPF2.1*-OE-2 plants. **(D)** The primary root length of indicated plants. **(E)** The Fv/Fm ratios of indicated plants before and after salt stress. **(F-G)** Accumulation of *in situ* H_2O_2 **(F)** and $O_2^{\cdot-}$ **(G)** content in indicated plants. The asterisks indicate significant differences as assessed by independent samples t-test, ***$P < 0.001$.

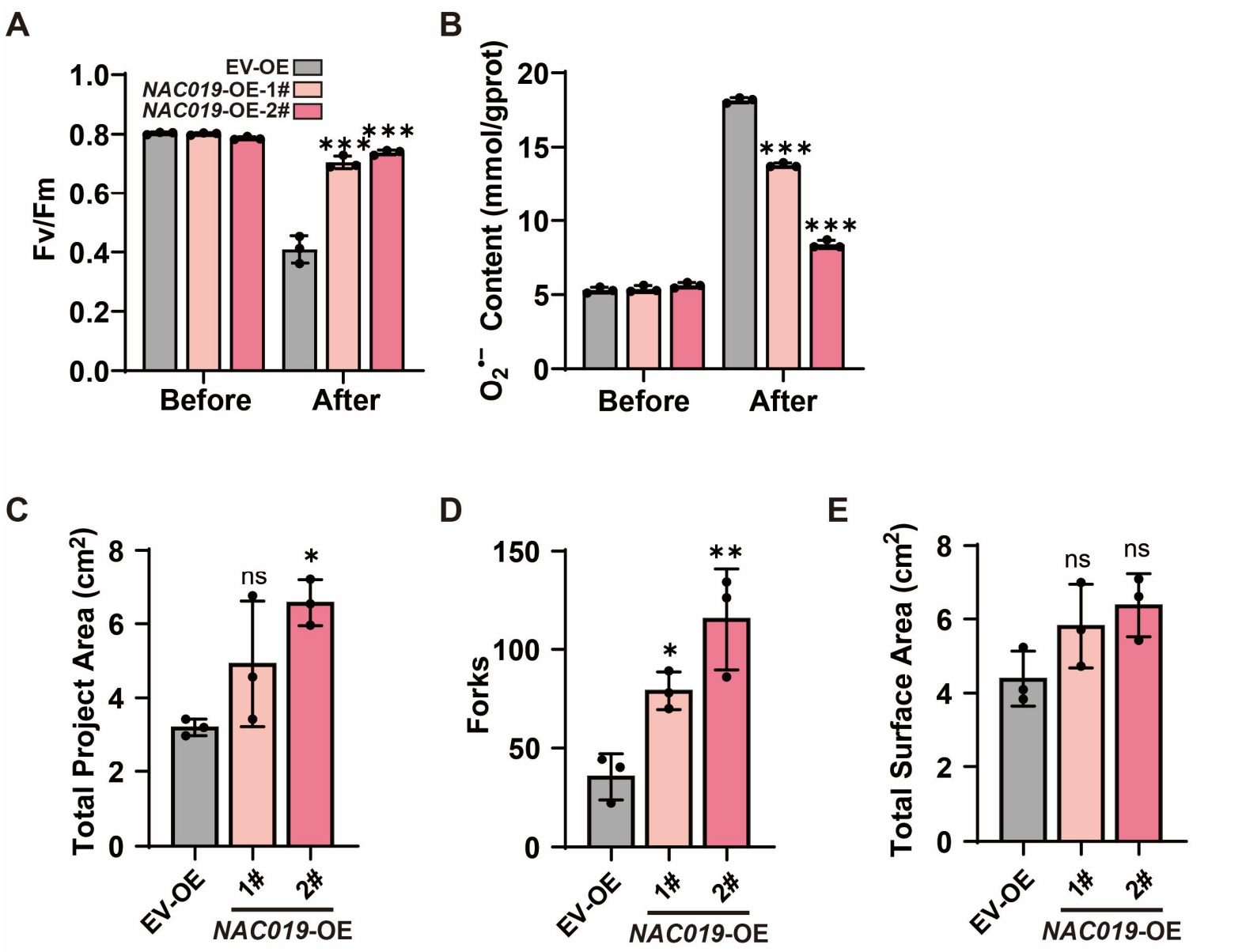

Supplementary Figure 15. The physiological experiment of *CtrNAC019*-OE plants. **(A)** The fluorescence of chlorophyll before and after treatment were detected response to Figure 6G. **(B)** The $O_2^{\bullet-}$ content in seedlings was measured before and after salt stress to quantify the degree of stress-induced damage. **(C-E)** The root parameter indicated the growth tendency conducting by root scanner, containing total project area **(C)**, forks **(D)**, and total surface area **(E)**. Values represent the mean ± SE with three biological replicates. The asterisks indicate significant differences as assessed by independent samples t-test, ns, $P > 0.05$, * 0.05< $P < 0.01$, ** 0.01< $P < 0.001$, ***$P < 0.001$.

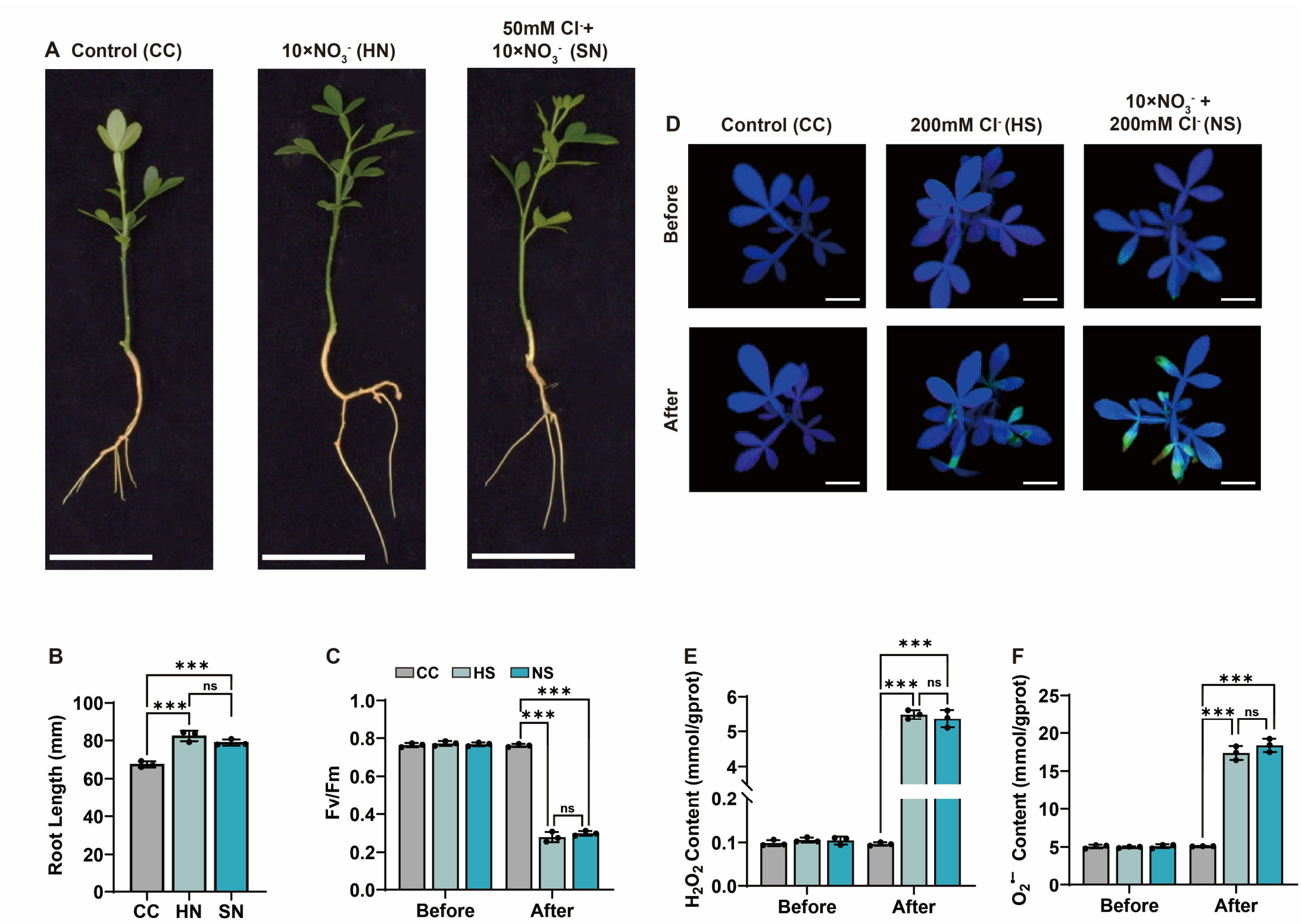

Supplementary Figure 16. The physiological experiment on salt-enhanced nitrate utilization (SENU) and nitrogen-mediated salt tolerance (NMST) of *CtrNPF2.1*-TRV plants. (A) Root photograph of 30th day during corresponding treatment. CC, normal control conditions. HN, 10-fold nitrate (50 mM NO_3^-) nutrition solution. SN, 50 mM NaCl pretreatment for 7 days and then transformed to 10-fold nitrate nutrition solution. Scale bars = 5 cm. **(B)** The primary root length of CC, HN, and SN in *CtrNPF2.1*-TRV plant. **(C-D)** Fv/Fm ratios **(C)** and chlorophyll fluorescence imaging **(D)** before and after corresponding treatment. Scale bars = 1 cm. CC, normal control conditions. HS, 200 mM NaCl with nutrition solution. NS, 50 mM KNO_3 pretreatment for 3 days and then transformed to 200 mM NaCl with nutrition solution. **(E-F)** Accumulation of in situ H_2O_2 **(E)** and $O_2^{\bullet -}$ **(F)** content in *CtrNPF2.1*-TRV plants. The asterisks indicate significant differences as assessed by independent samples t-test, ns, $P > 0.05$, * $0.05 < P < 0.01$, ** $0.01 < P < 0.001$, ***$P < 0.001$.
