# Supplementary material for: The Dual‐Function of CtrNAC019‐CtrNPF2.1 Module in Salt Tolerance and Nitrogen Use Efficiency Via Enhancing Vacuolar Chloride Sequestration and Nitrate Efflux in Citrus trifoliata
Source: Plant Biotechnol J. 2026 May 18:10.1111/pbi.70686. Online ahead of print. doi: 10.1111/pbi.70686 (PMC13398927; doi:10.1111/pbi.70686)
Supplement: Supplementary file 2 — Table S1: List of primers used in this study. [file PBI-9999-0-s002.docx]

**Supplementary Table 1**. List of primers used in this study
